# A metagenomic analysis of urban river samples reveals high numbers of sequences related to mycoviruses

**DOI:** 10.1007/s00705-025-06496-y

**Published:** 2026-01-08

**Authors:** Roland Zell, Marco Groth, Lukas Selinka, Hans-Christoph Selinka

**Affiliations:** 1 Section Experimental Virology, Institute for Medical Microbiology, Jena University Hospital Friedrich Schiller University, Hans-Knöll-Str. 2, 07745 Jena, Germany; 2 CF Next Generation Sequencing, Leibniz Institute on Aging, Fritz Lipmann Institute (FLI), Jena, Germany; 3 Section II 1.4 Microbiological Risks, Department of Environmental Hygiene, German Environment Agency, Berlin, Germany

## Abstract

Mycoviruses have been classified into 35 virus families so far. In addition to numerous mycoviruses with proven fungal or oomycetes hosts, many newly discovered viruses from environmental water and soil samples and various animal or plant specimens exhibit significant similarity to classified mycoviruses, thereby expanding the known sequence space of fungal and related viruses. In this study, we searched for mycoviruses in two environmental water samples that had been collected from the Teltow Canal and the Havel River in Berlin, Germany. Viral sequences with similarity to members of 16 virus families were identified. The most common viruses in our samples were botourmia-like viruses with moderate similarity to members of the genus *Ourmiavirus*. Notably, 58 of the ourmia-like sequences from the Teltow Canal and Havel River and 10 ourmia-like sequences from other sources exhibited a dicistronic genome layout. The second open reading frame (ORF) of these viruses encoded a putative capsid protein with an S domain that showed little similarity to the structural proteins of the classified ourmiaviruses. The second-largest virus group (59 sequences) was assigned to the order *Ghabrivirales*, and 13 of these sequences exhibited similarity to members of the suborder *Alphatotivirineae* (families *Orthototiviridae*, *Pseudototiviridae*, *Botybirnaviridae*, and *Chrysoviridae*). Thirty-three sequences clustered with members of the suborder *Betatotivirineae* – three of them with members of the family *Artiviridae*. Fifteen highly divergent toti-like sequences suggest the need to establish a new higher-order taxon within the order *Ghabrivirales*. Other virus sequences were assigned to the families *Mitoviridae* (three unuamitoviruses and 10 highly divergent mitovirus-like sequences), *Narnaviridae* (five "alphanarnavirus"-like sequences), *Amalgaviridae* (two zybavirus-like sequences), *Hypoviridae* (one partial RdRP sequence), and *Mymonaviridae* (one partial RdRP sequence), and one was not classified (Sclerophthora macrospora B-like virus). Notable results include a clade of highly divergent mitovirus-like sequences with a standard translation code, three narnavirus-like sequences with a reverse-frame ORF, and a clade of four *Ghabrivirales*-like replicase sequences that were found to have numerous termination codons regardless of which translation table was used.

**Supplementary Information:**

The online version contains supplementary material available at 10.1007/s00705-025-06496-y.

## Introduction

Mycoviruses have been detected in a wide range of host species comprising all of the major taxa of fungi and oomycetes. They exhibit great diversity in their genome structure and replication mechanisms. Moreover, mycoviruses have mostly nonlytic life cycles, as they forgo extracellular routes of infection and make no use of vectors but instead establish persistent asymptomatic infections of their fungal hosts. Mycoviruses propagate by efficient vertical and horizontal transmission routes. Typical vertical transmission routes are cell division and sporogenesis; horizontal transmission includes cell fusion mechanisms such as hyphal anastomosis and heterokaryosis [1]. Although viral persistence is common, mycoviruses with icosahedral particles have been described. The complex interactions of most mycovirus–fungus–plant and mycovirus–fungus–animal host systems are still underexplored, as is the ecological role of viral hyperparasitism [2]. Viral infection of some fungi may cause hyper- or hypovirulence of their plant-pathogenic hosts, with the latter feature making them potential viral agents for controlling virulent pathogenic fungi. Furthermore, some viruses can shuttle between fungi and plants or insects [3–5], and fungi may serve as vectors of plant viruses [6, 7].

Aquatic systems are often overlooked as fungal habitats although fungi play important roles in cycling of organic matter and food web dynamics [8]. Moreover, mycoviruses are also important players in these interactions and in ecological control [9]. In addition to their complex ecological role, the great genetic diversity of mycoviruses has attracted the attention of researchers in recent years. Hundreds of mycoviruses belonging to at least 35 virus families have been classified (see Virus Metadata Resource MSL40.v1.20250307, available for download from https://ictv.global/), but thousands of mycovirus-like sequences still await classification. Mycoviral genomes may consist of single-stranded DNA (*Genomoviridae*), segmented or unsegmented double-stranded RNA or single-stranded RNA with positive- or negative-strand polarity. Furthermore, positive-stranded RNA viruses with reverse transcriptase activity have also been described (*Metaviridae*, *Pseudoviridae*). Other oddities in the mycovirus world include single-stranded RNA viruses with circular genomes and two open reading frames in ambisense orientation (viruses of the phylum *Ambiviricota*) and multisegmented positive-stranded RNA viruses with a composite RNA-dependent RNA polymerase (RdRP) complex assembled from two separate domains that are encoded by different RNA segments (viruses of the family *Splipalmiviridae*) [10]. One mycovirus with a double-stranded DNA genome, rhizidiovirus, has been reported, but no sequence data are available [11] and its significance is unclear.

The number of published mycovirus sequences has increased exponentially with the availability of high-throughput sequencing techniques, and virus taxonomists have to cope with the continuously growing amount of sequence data and its classification. Each new published fungal metagenome and each new sequenced virome of environmental samples has revealed novel mycoviruses or mycovirus-like sequences [10, 12, 13]. Often, fungi may contain many different viruses. For example, the facultative plant-pathogenic fungus *Rhizoctonia solani* − which is in fact a complex of many related species, so-called anastomosis groups − hosts as many as 100 different viruses belonging to 18 virus families [14]. Similarly, the oomycete *Bremia lactucae* hosts more than 15 viruses of various families [15]. The situation is complicated by cross-kingdom infections, e.g., if fungi serve as vectors for plant viruses, or if viruses shuttle between fungi and plants or between fungi and insects [3–7, 16].

The Teltow Canal and Havel River are two waterbodies traversing the metropolitan area of Berlin, Germany. In our previous investigations of their fluvial viromes, we identified complete or partial genomes of more than 1500 novel RNA viruses, which were reported in a series of papers [17–21]. Here, we add viral sequences detected in samples from these waterbodies that are related to known mycoviruses. The novel viruses exhibit similarity to members of the suborders *Alpha*- and *Betatotivirineae* and the families *Botourmiaviridae*, *Mitoviridae*, *Narnaviridae*, and *Amalgaviridae* (genus *Zybavirus*), as well as to the unclassified Sclerophthora macrospora B virus.

## Materials and methods

Sample collection and methods of virus enrichment, RNA extraction, Illumina sequencing, sequence data processing, and sequence analysis have been described previously [21]. They are briefly summarized here:

The 50-L Teltow Canal water sample (ID MR233-17) was collected in Berlin, Bäkebrücke (site coordinates: 52°26′03″ N, 13°18′57″ E), on July 18, 2017. The 50-L Havel River water sample (ID MR644-17) was collected in Berlin, Heerstrasse (site coordinates: 52°30'46'' N 13°12'14'' E), on June 28, 2018. Both 50-L samples were divided into five 10-L aliquots and further treated as described by Wyn-Jones et al. [22]. For this, the aliquots were stirred for 20 minutes to suspend floating detritus, acidified to pH 3.5 by addition of hydrochloric acid, and loaded on a glass-wool-packed column for adsorption of virus particles. Adsorbed virus particles were consecutively washed with hydrochloric acid (1 M), sodium hydroxide (1 M), and tap water and finally eluted with 3% beef extract/0.05 M glycine buffer, pH 9.5. Eluates were neutralized by addition of sodium hydroxide and passed through a 0.45-µm filter to remove residual detritus and bacteria. Virus particles were sedimented by ultracentrifugation (100,000 × *g*, 2.5 h at 4 °C). Finally, the sediment was redissolved in 0.5 ml of phosphate-buffered saline by agitation in a ball mill and stored at −80°C for later RNA extraction.

RNA was extracted using a QIAamp Viral RNA Mini Kit (QIAGEN, Hilden, Germany) according to the manufacturer's instruction and analyzed by Illumina sequencing as described by Zell et al. [21]. Briefly, 100 ng of RNA from the Havel River sample was used for library preparation employing an Illumina TruSeq Stranded Total RNA Library Preparation Kit, whereas the library from the Teltow Canal sample was prepared from 450 ng of RNA using an Illumina TruSeq Stranded Total RNA Library Preparation Kit combined with a Ribo-Zero Gold rRNA Removal Kit according the manufacturer’s instructions. The protocol was adapted to include all RNA molecules rather than only polyadenylated RNA by adding an RNA precipitation step with isopropanol and redissolving the pellet in Fragment, Prime, Finish Mix (FPF). Thereafter, library quantitation and quality checking were done using a DNA 7500 kit and a 2100 Bioanalyzer instrument (Agilent Technologies, Waldbronn, Germany). Paired-end Illumina sequencing (2 × 150 bp) was done on a HiSeq 2500 platform using the rapid run mode.

Sequence data were extracted in FastQ format using bcl2FastQ v2.19.1.403 (Illumina). Adapter sequences were removed using Cutadapt v1.8.3 [23]. After duplicon extraction, the remaining 70,018,635 read pairs of the Teltow Canal sample and 51,902,006 read pairs of the Havel River sample were used for *de novo* assembly by two methods: (i) Employing clc_assembler v5.2.1 (QIAGEN) with the parameters -p fb ss 50 500, a total of 537,529 contigs longer than 200 nucleotides were obtained from the Teltow Canal sample, and 162,082 contigs were obtained from the Havel River sample. (ii) Employing the metaSPAdes assembler v3.15.3 [24] with standard parameters (-k auto), 1,314,849 scaffolds were obtained from with the Teltow Canal sample, and 388,367 were obtained from the Havel River sample. The final sequences were obtained by manual curation, i.e., linking of overlapping contigs and scaffolds.

We used a 2-step procedure for analysis of sequence data. First, virus-specific scaffolds and contigs were identified by comparing the assembled sequences with our in-house virus protein database compiled from all NCBI GenBank entries with the Taxonomy ID 10239, using DIAMOND v2.0.10 [25]. DIAMOND identified 66,371 virus-specific scaffolds and 41,408 contigs from the Teltow Canal sample and 16,922 scaffolds and 8810 contigs from the Havel River sample. In the second step, selected DIAMOND hits were confirmed using BLAST+ v2.13.0 (https://ftp.ncbi.nlm.nih.gov/blast/executables/blast+/2.13.0/) with the BLASTp and tBLASTx tools. If appropriate, specific BLASTn searches were performed with reference sequences downloaded from GenBank. For identification of conserved protein domains, the NCBI web search tools BLASTp suite and Pfam conserved domain database (CDD) were used (https://blast.ncbi.nlm.nih.gov/Blast.cgi, https://www.ncbi.nlm.nih.gov/Structure/cdd/wrpsb.cgi). ClustalW and MUSCLE implemented in MEGA X [26] were employed for protein sequence alignments. Alignments were adjusted manually if necessary. For construction of phylogenetic trees, we used IQ-TREE 2.1.3 for Windows [27]. The best-fit substitution models were identified using the automatic model selection option (ModelFinder) implemented in IQ-TREE. Branch support was assessed using the ultrafast bootstrap approximation UFBoot2 with 10,000 replications [28].

All viruses were provisionally assigned to established taxa using the current virus taxonomy (2024 release) based on the most recent Master Species List #40 (https://ictv.global/msl; accessed on March 7, 2025) and the most recent Virus Metadata Resource spreadsheet MSL40.v1.20250307 (https://ictv.global/vmr/current; released on March 7, 2025).

## Results

Analysis of the Teltow Canal and the Havel River viromes revealed the presence of numerous RNA viruses with similarity to fungal viruses. Genomic sequences related to members of 15 virus families known to contain mycoviruses were detected (Table 1). These included – in alphabetical order *Alphaflexiviridae*, *Amalgaviridae*, *Artiviridae*, *Barnaviridae*, *Botybirnaviridae*, *Botourmiaviridae*, *Chrysoviridae*, *Endornaviridae*, *Hypoviridae*, *Mitoviridae*, *Mymonaviridae*, *Narnaviridae*, *Orthototiviridae*, *Partitiviridae*, and *Pseudototiviridae*. In addition, sequences related to the unclassified Sclerophthora macrospora viruses A and B and Plasmopara halstedii virus A, as well as many unclassified viruses of the orders *Ghabrivirales* and *Cryppavirales* were detected. Furthermore, viruses with similarity to fungus-associated aspivirus-like viruses were identified (Table 1). Notably, the majority of the novel virus sequences exhibited similarity to members of the family *Botourmiaviridae* (positive-strand RNA viruses) or the order *Ghabrivirales* (double-strand RNA viruses), but most of the new sequences were highly divergent compared to those of the established members. As Teltow Canal and Havel River viruses with similarity to alphaflexiviruses, barnaviruses, endornaviruses, and partitiviruses have been described previously [19], they were excluded here. No significant sequences corresponding to mycoviruses of the families *Polymycoviridae* (dsRNA), *Deltaflexiviridae* (+RNA), *Fusariviridae* (+RNA), *Gammaflexiviridae* (+RNA), *Hadakaviridae* (+RNA), *Yadokariviridae* (+RNA), *Discoviridae* (–RNA), *Phenuiviridae* (–RNA), *Tulasviridae* (–RNA), *Metaviridae* (RT viruses), or *Pseudoviridae* (RT viruses) were found.

**Table 1:** Viral sequences from the Teltow Canal and Havel River with similarity to sequences of mycoviruses

Phylum	Order	Family	No. of sequences	Genome	Reference
*Pisuviricota*	*Durnavirales*	*Amalgaviridae*	2	dsRNA	This study
		*Partitiviridae*	12	dsRNA	[19]
		*Hypoviridae*	1	+RNA	This study
	*Sobelivirales*	*Barnaviridae*	1	+RNA	[19]
*Duplornaviricota*	*Ghabrivirales*	*Artiviridae*	4	dsRNA	This study
		*Botybirnaviridae*	4	dsRNA	This study
		*Chrysoviridae*	3	dsRNA	This study
		*Pseudototiviridae*	4	dsRNA	This study
		*Orthototiviridae*	2	dsRNA	This study
		[*Ghabrivirales* sp.]	42	dsRNA	This study
*Kitrinoviricota*	*Martellivirales*	*Endornaviridae*	20	+RNA	[19]
	*Tymovirales*	*Alphaflexiviridae*	28	+RNA	[19]
*Lenarviricota*	*Ourlivirales*	*Botourmiaviridae*	139	+RNA	This study
	*Cryppavirales*	*Mitoviridae*	3	+RNA	This study
		[*Mitoviridae* sp.]	10	+RNA	This study
	*Wolframvirales*	*Narnaviridae*	5	+RNA	This study
*Negarnaviricota*	*Mononegavirales*	*Mymonaviridae*	1	–RNA	This study
	*Naedrevirales*	*Aspiviridae*	4*	–RNA	[19]
Unclassified	Plasmopara halstedii virus A-like		1	+RNA	This study
	Sclerophthora macrospora virus A-like		45	+RNA	This study, [19]
	Sclerophthora macrospora virus B-like		1	+RNA	This study

* similarity to fungus-associated aspi-like viruses

**Fig. 1:**
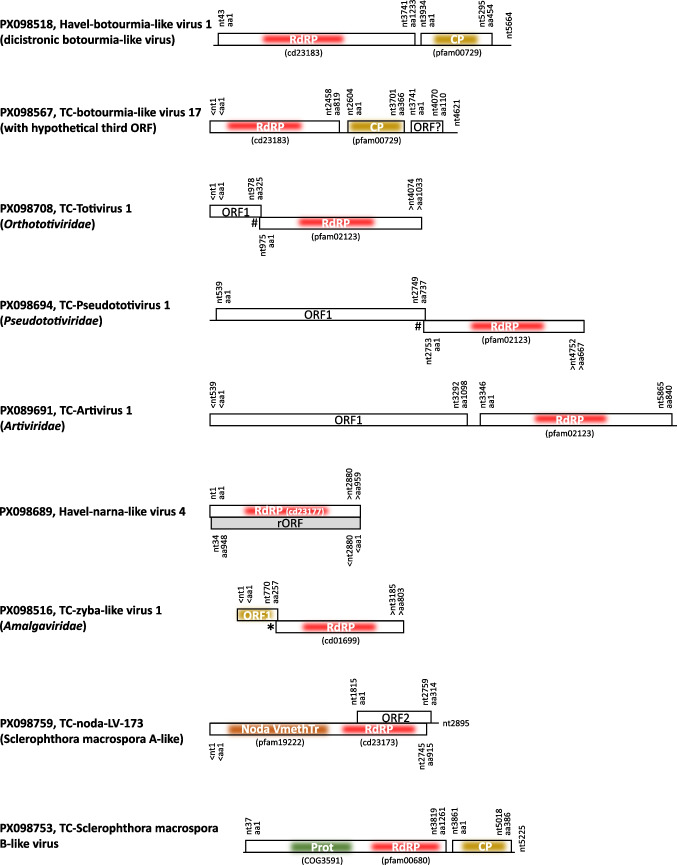
Genome layout of selected novel viruses from the Teltow Canal and Havel River. ORFs are indicated by boxes. Locations of conserved protein domains identified using the Conserved Domain Database (CDD) search tool are indicated. Abbreviations: CP, capsid protein (highlighted in ochre); ORF, open reading frame; Prot, V8-like Glu-specific endopeptidase (COG3591) (green); RdRP, RNA-dependent RNA polymerase (red). An asterisk (*) indicates the presence of a ribosomal frameshift site (UUUCUUA) at nt position 750–756 of TC-zyba-like virus 1, whereas hashtags (#) indicate stop-restart sites (AUGA) in TC-totivirus 1 and TC-pseudototivirus 1, respectively. Nucleotide and amino acid positions are indicted

The most characteristic features of the detected mycovirus-like sequences and the high variability in their genomic layouts (Fig. 1) are outlined in the following paragraphs:

### *Botourmiaviridae*

The majority of the sequences obtained in this study were related to botourmiaviruses. Viruses of the family *Botourmiaviridae* (order *Ourlivirales*) infect plants, fungi, and possibly oomycetes. The family comprises 12 genera, one of which is the genus *Ourmiavirus*, whose members have a positive-stranded RNA genome with three monocistronic segments, produce non-enveloped bacilliform virions, and infect plants [29]. The viruses of the remaining genera have non-encapsidated monopartite, monocistronic positive-stranded RNA genomes and infect fungi. The sizes of the monopartite genomes of the classified botourmiaviruses range from 2 to 5 kb. The gene segments of the tripartite ourmiaviruses are 2.8 kb, 1.1 kb, and 1 kb long. The RNA of fungus-infecting botourmiaviruses and RNA segment 1 of ourmiaviruses encode a replicase (cd23183), whereas RNA segment 2 of ourmiaviruses encodes a movement protein, and segment 3 encodes a coat protein with an S domain (pfam00729). The RdRP sequences of all botourmiaviruses cluster with those of leviviruses (now assigned to the family *Fiersviridae*), mitoviruses, and narnaviruses on branch 1 of the so-called 'comprehensive RdRP tree' [30]. The viruses of this branch have recently been assigned to the phylum *Lenarviricota* [31].

DIAMOND identified 121 sequences from the Teltow Canal sample and 33 from the Havel River sample encoding a botourmia-like RdRP (cd23183). These sequences were named TC-botourmia-LVs and Havel-botourmia-LVs. Notably, five clades contained dicistronic virus sequences. Altogether, 68 viruses exhibited a dicistronic genome layout with ORF 1 encoding the RdRP (cd23183) and ORF 2 encoding a putative capsid protein with an S domain (pfam00729). The genome of TC-botourmia-LV-17 has a putative third ORF with unknown significance. Another sequence of 2589 nt was identified by the CDD tool as a botourmia-like virus based on a short stretch of 120 amino acids representing a partial RdRP sequence (cd23183), although with low statistical significance (1.31e-12). This RdRP sequence was located on the C-terminal side of a helicase domain, which is unusual for botourmiaviruses.

The RdRP sequences of the botourmia-like viruses were aligned with the respective sequences of classified reference viruses, including a number of mitoviruses and narnaviruses as an outgroup. The phylogenetic tree reproduced three branches corresponding to the families *Botourmiaviridae*, *Mitoviridae*, and *Narnaviridae* (Supplementary Fig. S1). None of the Teltow Canal or Havel River botourmia-like viruses clustered with members of any of the fungus-infecting viruses but were related to plant-infecting ourmiaviruses.

**Fig. 2:**
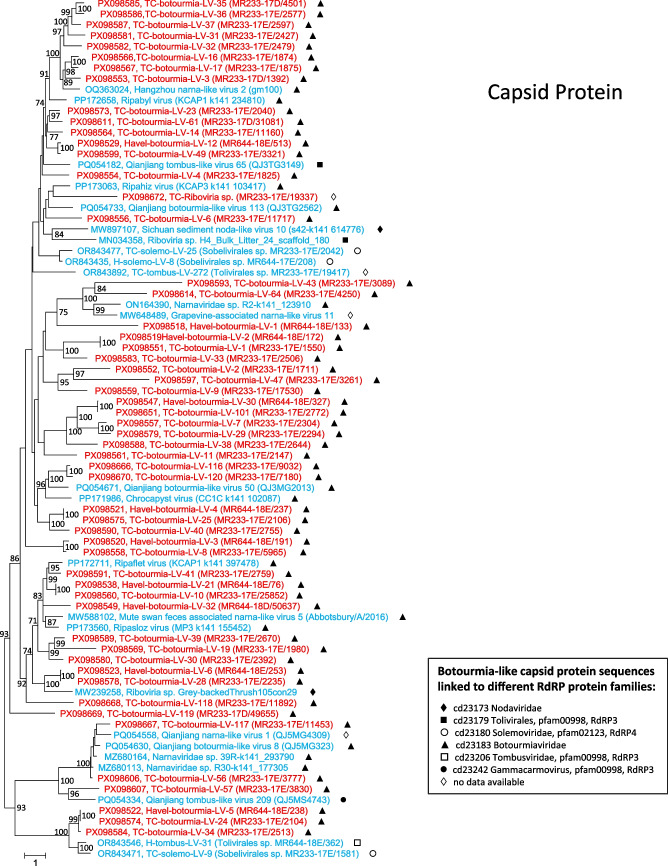
Phylogenetic analysis of 80 capsid protein sequences of members of dicistronic unclassified botourmia-like viruses. The CP sequences of members of five clades of botourmia-like viruses from the Teltow Canal and Havel River (listed in Supplementary Figure S1) were aligned with CP sequences from various hosts and used for construction of a maximum-likelihood tree, using IQ-TREE 2 (optimal substitution model: Q.pfam+F+R5). Presented are GenBank accession numbers, vernacular virus names, and strain designations, if available (in parentheses). Numbers at nodes indicate bootstrap support greater 65% obtained after 10,000 ultrafast replications. The bar indicates amino acid substitutions per site. Color code: blue, unclassified viruses; red, viruses from the Teltow Canal and Havel River. Symbols (♦, ◼, ○, ▲, ◻, ●, ♢) indicate the CDD family of the corresponding RdRP

To examine the sequences of the structural proteins, we aligned these sequences with the most similar CP sequences identified using BLAST, but this failed to yield a significant alignment with the ourmiavirus CP sequences. A CDD search of the polymerase sequences linked to the CP sequences shown in the phylogenetic tree in Fig. 2 revealed six protein families, i.e. cd23183 (Botourmiaviridae-RdRP, ▲), cd23173 (Nodaviridae-RdRP, ♦), cd23179 (Tolivirales-RdRP, ◼), cd23180 (Solemoviridae-RdRP, ○), cd23206 (Tombusviridae-RdRP, ◻) and cd23242 (Gammacarmovirus-RdRP, ◻). This finding might suggest that recombination events occurred frequently in the evolution of these RNA viruses.

### *Ghabrivirales*

The taxonomy of the order *Ghabrivirales* is based on the polymerase phylogeny, resulting in three suborders, *Alpha*-, *Beta*- and *Gammatotivirineae*, which together include 19 virus families [31]. Monopartite and multipartite double-stranded RNA genomes with variable gene layouts have been described in this order. The polymerase belongs to the protein family pfam02123 (RdRP4). Where structural data are available, the members of the *Ghabrivirales* have been shown to possess an icosahedral capsid with a size of 35–50 nm. The capsid protein gag of L-A virus (species *Totivirus ichi*, family *Orthototiviridae*) which is the best-studied member of the order *Ghabrivirales*, belongs to protein family pfam09220 and exhibits similarity to the reovirus capsid protein regarding protein folding and arrangement in a T=1/pseudo T=2 capsid symmetry [32].

We investigated 59 sequences from the Teltow Canal and Havel River samples with similarity to members of the order *Ghabrivirales*. Our alignment of RdRP sequences includes reference sequences of all 19 *Ghabrivirales* families plus several unclassified viruses. The resulting tree supports the present taxonomy (Supplementary Fig. S2). Three branches correspond to the three suborders. All 19 families exhibit long branch lengths. Within the suborder *Alphatotivirineae*, two new orthototiviruses, four new pseudototiviruses, and four new botybirna-like viruses were identified. Noteably, our orthototi- and pseudototiviruses possess overlapping stop/start codons (UAAUG) at the ORF1-ORF2 junction, which would enable a coupled termination-reinitiation mechanism for the expression of the capsid protein and RdRP rather than a programmed ribosomal shift. Furthermore, 33 toti-like viruses, including three artiviruses, grouped with members of the suborder *Betatotivirineae*. Fifteen toti-like viruses, which clustered distinctly from the three known suborders, were also observed. This clade of divergent viruses suggests the need for another higher-order taxon within the order *Ghabrivirales*. Among these divergent viruses, one subclade has four RdRP-like sequences that contain many internal stop codons regardless of which translation table is used. The significance of this finding remains to be elucidated.

In addition, DIAMOND identified three scaffolds representing alphachrysoviruses. One 278-nt scaffold corresponded to segment 1 (RdRP) and exhibited 52% aa sequence identity to Brassica campestris chrysovirus 1 (accession no. KP782031). However, this fragment was too short for a proper phylogenetic analysis. Two other scaffolds with lengths of 671 and 546 nt, respectively, showed about 30% aa sequence identity to the major capsid proteins of two different alphachrysoviruses (data not shown). No *Ghabrivirales* sequences with significant similarity to those of members of the *Alternaviridae*, *Fusagraviridae*, *Giardiaviridae*, *Inseviridae*, *Lebotiviridae*, *Megabirnaviridae*, *Megatotiviridae*, *Monocitiviridae*, *Ootiviridae*, *Phlegiviridae*, *Pistolviridae*, *Quadriviridae*, *Spiciviridae*, or *Yadonushiviridae* were found.

### *Narnaviridae* and *Mitoviridae*

Narnaviruses and mitoviruses possess a non-encapsidated positive-stranded RNA genome ranging from 2.1 to 5 kb in length. Its single ORF encodes an RdRP. A recent taxonomic revision removed mitoviruses from the family *Narnaviridae* and placed them into the newly created family *Mitoviridae* based on the low degree of similarity of their replicase sequences. The family *Narnaviridae* (order *Wolframvirales*) is comprised of a single genus with two species. In contrast, the family *Mitoviridae* (order *Cryppavirales*) includes four genera with 105 species [33]. Numerous related viruses from a wide spectrum of hosts await classification. Whereas the two acknowledged narnaviruses infect yeast cells, most mitoviruses use filamentous fungi as hosts and utilize translation table 4, in which UGA encodes tryptophan, indicating persistence in fungal mitochondria [34]. Only a few mitoviruses have been associated with plant hosts [35, 36], and those viruses use the standard genetic code.

By comparing RdRP sequences, we identified five sequences with similarity to those of narnaviruses (cd23177) and 13 with similarity to those of mitoviruses (pfam05919). Phylogenetic analysis of these RdRP sequences revealed that all of the narna-like viruses were novel and clustered with viruses that were recently proposed to be classified as "alphanarnaviruses" [37] (Supplementary Fig. S3). Notably, three viruses of the "alphanarnavirus" clade (TC-narna-LV-1 and Havel-narna-LV-3 and −4) contained a reverse-frame ORF (rORF, also known as an ambigrammatic sequence), as has been described previously for some narna-like viruses [37–40].

The *Mitoviridae* branch contains monophyletic clades of the four mitovirus genera, albeit with moderate bootstrap support (Supplementary Fig. S3). Three of our mito-like sequences clustered with unuamitoviruses (Havel-mito-LV-4, Havel-mito-LV-5, and TC-mito-LV-8). The remaining mito-like sequences from the Havel River and Teltow Canal were too divergent to assign them to an existing mitovirus genus. Two clades of the tree contain viruses which utilize the standard translation table rather than translation table 4. One of these clades comprises nine plant-infecting mitoviruses of the genus *Duamitovirus*. The other clade consists of a group of eight novel virus sequences from the Teltow Canal and Havel River plus six still unclassified "narna-like" viruses that have been proposed recently to be members of the order *Cryppavirales* [33]. These six "narna-like" viruses were associated with hosts as diverse as plants, insects, tunicates, penguins, and rabbits. The use of the standard translation table is compatible with the reported non-fungal hosts and also suggests non-fungal hosts for the eight Teltow Canal and Havel River viruses of this clade. Another novel, untypeable virus is Havel-mito-like virus 3, which also lacks internal UGA codons, suggesting the use of the standard genetic code.

### *Amalgaviridae*

The family *Amalgaviridae* (order *Durnavirales*) consists of three genera: *Amalgavirus*, with plant-infecting viruses; *Unirnavirus*, with viruses of filamentous ascomycetes; and *Zybavirus*, with viruses that infect budding yeast. Members of the *Amalgaviridae* have double-stranded RNA genomes with two open reading frames. ORF 1 encodes a protein of unknown function and is not conserved among the genera. The second ORF is partly overlapping with ORF 1 and is expressed as a fusion protein, either by a –1 or a +1 ribosomal frameshift [41]. The fusion protein is a polyprotein with an RdRP1 domain (pfam00680, cd01699). Members of the *Amalgaviridae* cause persistent infections in their hosts without a visible cytopathic effect.

**Fig. 3:**
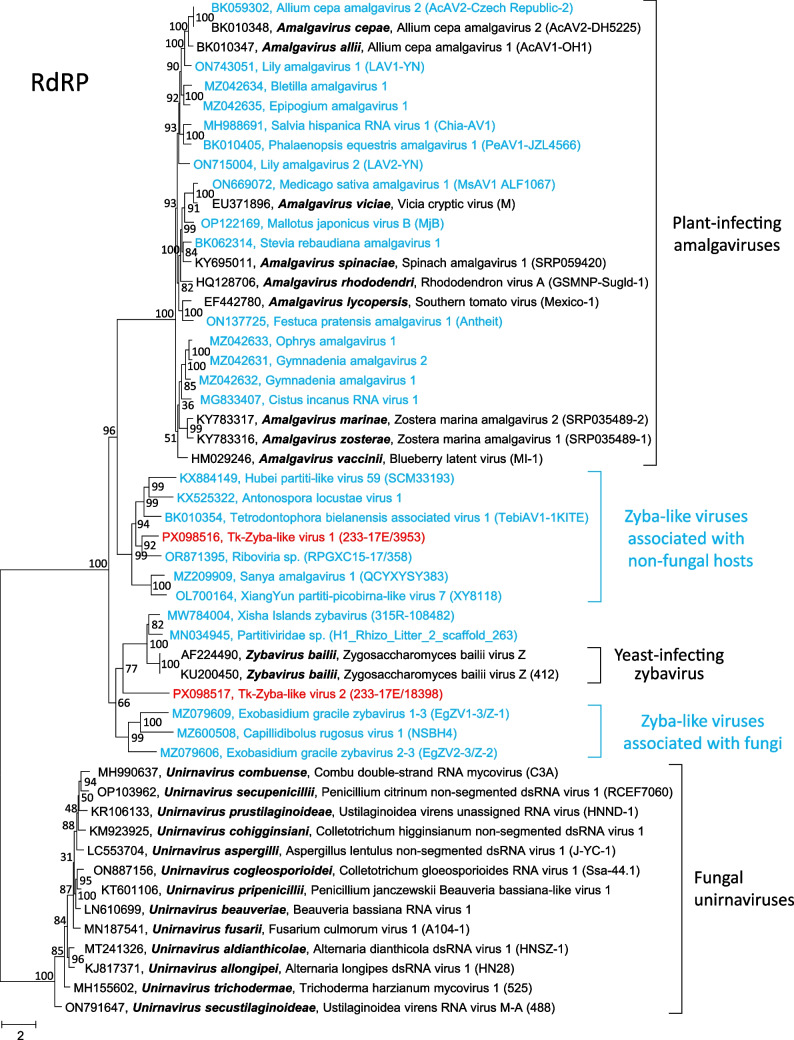
Phylogenetic analysis of 52 RdRP sequences of members of the family *Amalgaviridae* and related viruses. The replicase sequences of members of all species of the *Amalgaviridae*, unclassified related viruses from the Teltow Canal, Havel River, and various hosts were aligned using MEGA, and a maximum-likelihood tree was constructed using IQ-TREE 2 with the optimal substitution model Q.pfam+F+R6. Presented are GenBank accession numbers, vernacular virus names, and strain designations, if available (in parentheses). Square brackets indicate hosts. Numbers at nodes indicate bootstrap support obtained after 10,000 ultrafast replications. The tree was arbitrarily rooted using unirnavirus sequences. The bar indicates amino acid substitutions per site. Color code: black, classified viruses; blue, unclassified viruses; red, viruses from the Teltow Canal and Havel River

DIAMOND identified two scaffolds with similarity to the zybaviruses. TC-zyba-like virus 1 has a length of 3185 nt and represents the nearly complete ORFs 1 and 2 (Fig. 1). In comparison to Antonospora locustae virus 1, an unclassified zyba-like virus (accession no. KX525322), the fusion protein lacks fewer than five amino acids at both the N- and C-terminal ends. The sequence of TC-zyba-like virus 2 corresponds to a partial RdRP. A phylogenetic analysis based on the RdRP sequences of all members of the *Amalgaviridae* plus several related but unclassified virus sequences, including both TC-zyba-like sequences, revealed three major clades corresponding to the three amalgavirus genera plus two clades with zyba-like virus sequences. One of these clades includes fungal viruses, while the other is comprised of viruses that are associated with non-fungal hosts (Fig. 3).

### Sclerophthora macrospora viruses A and B, Plasmopara halstedii virus A

The oomycete *Sclerophthora macrospora* is an important pathogen that causes downy mildew disease of cereal crop plants of the family Poaceae. Two viruses infecting *S. macrospora* have been isolated so far: Sclerophthora macrospora viruses A and B (SmVA and SmVB). A third oomycete-infecting virus is Plasmopara halstedii virus A (PhVA). These three viruses have icosahedral capsids with sizes of 32 nm (SmVA), 35 nm (SmVB), and 37 nm (PhVA), respectively [42–44]. SmVA and PhVA have segmented single-stranded RNA genomes with RNA segment 1 encoding noda-like methyltransferase (pfam19222) and RdRP (cd23173) domains, whereas RNA segment 2 codes for a capsid protein with similarity to tombusviruses (pfam00729) [45, 46]. In addition, SmVA possesses a third satellite-like RNA. In contrast, the genome of SmVB consists of a monopartite, dicistronic single-stranded RNA (Fig. 1). The polyprotein of SmVB that is encoded by ORF 1 includes a V8-like Glu-specific endopeptidase, several VPg oligopeptides, and a polymerase that is related to the RdRPs of solemoviruses [47]. The second ORF codes for a capsid protein.

**Fig. 4:**
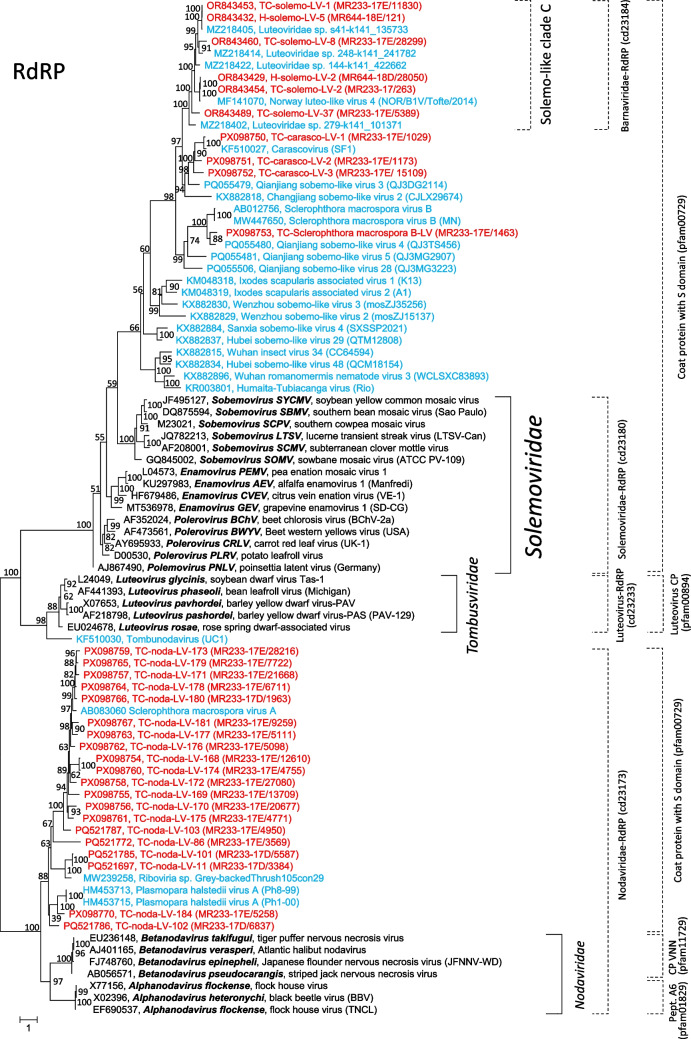
Phylogenetic analysis of 85 RdRP sequences of oomycetes viruses, related viruses, and reference viruses. The replicase sequences of Sclerophthora macrospora viruses A and B, Plasmopara halstedii virus A, unclassified related viruses from the Teltow Canal, Havel River, and various hosts as well as reference viruses (nodaviruses, luteoviruses, and solemoviruses) were aligned using MEGA, and a maximum-likelihood tree was constructed using IQ-TREE 2 with the optimal substitution model VT+F+R5. Presented are GenBank accession numbers, vernacular virus names, and strain designations, if available (in parentheses). Square brackets indicate family names (solid lines) and protein families of polymerase and coat proteins according to the CDD and pfam databases (broken lines). Numbers at nodes indicate bootstrap support obtained after 10,000 ultrafast replications. The tree was arbitrarily rooted with sequences with a *Nodaviridae* RdRP (cd23173). The bar indicates amino acid substitutions per site. Color code: black, classified viruses; blue, unclassified viruses; red, viruses from the Teltow Canal and Havel River

**Fig. 5:**
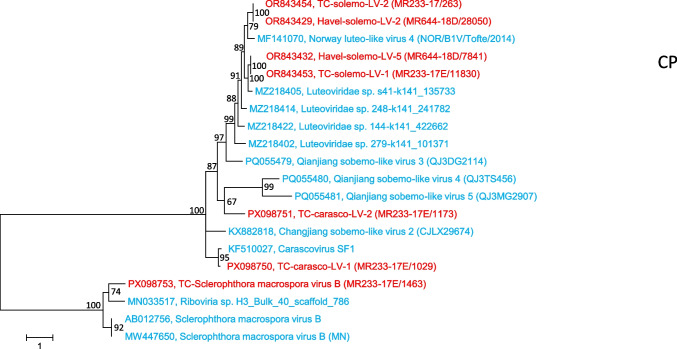
Phylogenetic analysis of 20 capsid protein sequences of Sclerophthora macrospora virus B and related viruses. The CP sequences of Sclerophthora macrospora virus B and unclassified related viruses from the Teltow Canal, Havel River, and various hosts were aligned using MEGA, and a maximum-likelihood tree was constructed using IQ-TREE 2 with the optimal substitution model: Q.pfam+F+G4. Presented are GenBank accession numbers, vernacular virus names, and strain designations, if available (in parentheses). Numbers at nodes indicate bootstrap support greater than 65% obtained after 10,000 ultrafast replications. The bar indicates amino acid substitutions per site. Color code: blue, unclassified viruses; red, viruses from the Teltow Canal and Havel River

Our phylogenetic analysis of the RdRP sequences of 85 viruses, including SmVA, SmVB, PhVA, solemoviruses, tombusviruses, nodaviruses, and a number of unclassified viruses, revealed six clades of unclassified viruses that clustered on the branch with the *Solemoviridae* members (Fig. 4, upper branch). Among these are (i) the previously described solemo-like clade C viruses with a barna-like RdRP (cd23184 [19]), (ii) carascovirus and similar viruses from the Teltow Canal, (iii) SmVB and related viruses, and (iv) three clades of Chinese solemo-like viruses from invertebrate samples. The viruses of this branch were characterized by the presence of an S domain (pfam00729) in their capsid protein. Phylogenetic analysis of the CP sequences of these six clades revealed the relatedness of their structural proteins (Fig. 5). However, our attempts to align the CP sequence of the detected TC-SmVB-like virus and *Solemoviridae* members failed. Too little similarity was observed (data not shown) although a CDD search suggested that both capsid proteins belong to protein family pfam00729. Notably, most of the viruses of the upper branch have a serine proteinase domain, either a trypsin-like proteinase (solemoviruses, viruses of the solemo-like clade C, carasco-like viruses, sobemo-like viruses from China) or a V8-like glutamyl endopeptidase, which is a characteristic of SmVB, the SmVB-like virus from the Teltow Canal, and Qianjiang sobemo-like virus 4.

The other oomycetes viruses, SmVA and PhVA, as well as 20 related viruses from the Teltow Canal clustered with nodaviruses, but in a distinct clade (Fig. 4 lower branch, see also reference 19).

### *Hypoviridae*

A single 264-bp scaffold related to a hypovirus was identified. It showed 47% aa sequence identity to Botrytis cinerea hypovirus 2, accession no. MN617169 (data not shown).

### *Mymonaviridae*

A 216-nt scaffold was assigned by DIAMOND to *Lentimonavirus lentinulae*, a species of negative-strand RNA viruses of the family *Mymonaviridae*. According to BLASTp, this sequence showed 57% aa sequence identity to the RdRP of Golovinomyces magnicellulatus associated virus and approximately 53% identity to the RdRPs of Erysiphe necator associated negative-stranded RNA virus 23 and Plasmopara viticola lesion associated mononegaambi virus 8 (data not shown).

## Discussion

In order to better understand the virome composition of riverine freshwaters, we examined the virus content of two 50-L water samples from the Teltow Canal and Havel River, which traverse the metropolitan area of Berlin, Germany. Both freshwater bodies are affected by human activity and contain microbes associated with municipal surface water drains, discharged effluents from a wastewater treatment plant, and recreational activities. Our previous work revealed the presence of thousands of novel, highly divergent virus sequences. In recent papers, we focused on picorna-like viruses, hepeliviruses, plant viruses, and invertebrate viruses [17–21], and these included descriptions of mycovirus-like sequences resembling sequences of members of the families *Alphaflexiviridae*, *Barnaviridae*, *Endornaviridae*, *Partitiviridae*, and *Spinareoviridae* and of the order *Elliovirales* [19, 21]. These virus families are known to include fungal viruses. Although environmental samples do not allow us to associate sequences with specific hosts, phylogenetic analysis suggested that the alphaflexiviruses, bunyaviruses, and spinareoviruses from the Teltow Canal and Havel River were not mycoviruses. In contrast, the SmVA- and PhVA-like viruses, endorna-like viruses, gammapartitiviruses, and aspi-like viruses were related to mycoviruses or still-unclassified fungus-associated viruses [19, 21]. Unfortunately, the possible hosts of the barna-like viruses and many of the mycovirus-related sequences in our present study remain uncertain because the related reference virus sequences are associated with both fungal and non-fungal hosts. This illustrates once more the limitations of using environmental samples. Among the detected and analyzed viruses whose hosts are still uncertain are the following viruses:

### TC- and Havel-botourmia-like viruses

Although we characterized more than 150 botourmia-like viruses, none of them belonged to the known genera of fungus-infecting viruses with monopartite, monocistronic genomes. Even their similarity to the plant-infecting ourmia viruses was only moderate. Five clades of botourmia-like viruses exhibited a dicistronic genome layout, suggesting that there are a number of new taxa to be created. Chen et al. and Sadiq et al. have also described botourmia-like viruses with dicistronic genomes [48, 49]. Moreover, GenBank has released a number of similar dicistronic botourmia-like virus sequences from other unpublished metagenomic studies. The presence of a second ORF encoding a putative CP with an S domain suggests that these viruses form virions with icosahedral or hemi-icosahedral capsids. At present, *Ourmiavirus* is the only genus of the family *Botourmiaviridae* whose members are known to have a capsid. Ourmiaviruses have a tripartite genome, with the genes for the RdRP, CP, and movement protein located on separate segments. Our data do not indicate whether the dicistronic botourmia-like viruses have a segmented genome or encode a movement protein, which would suggest a plant host. Qianjiang botourmia-like virus 13 (PQ054635) and TC-boturmia-like virus 17 both have a third ORF, but because no information is available on the expression or function of these hypothetical proteins, the significance of the additional ORF is unclear.

### *Ghabrivirales*-like viruses from the teltow canal and havel river

The association of 59 viruses with similarity to members of the order *Ghabrivirales* with their possible hosts remains inconclusive. Some artiviruses, orthototiviruses, and pseudototiviruses are known to infect both fungi and plants. The known botybirnaviruses exhibit specificity for fungi, but our TC-botybirna-like viruses are only distantly related to them and may utilize other hosts. Our remaining viruses of the order *Ghabrivirales* either belong to the suborder *Betatotivirineae* (*n =* 30), which includes only a few mycoviruses, or are too divergent to be assigned to any of the existing *Ghabrivirales* suborders, suggesting the need to create a new higher order taxon (*n =* 15). Related viruses of this clade for which host data are available do not appear to be associated with fungi. Nevertheless, this clade contains a branch with four interesting sequences with numerous in-frame termination codons, regardless of which translation table is used. It remains to be determined whether these sequences belong to endogenous, inactive virus-like RNA or indicate the existence of viruses that use a genetic code for protein translation that has not yet been described. Another possible explanation for this phenomenon could be that their genomes contain unusual RNA base modifications that lead to misincorporations during the reverse transcription step of library preparation.

### TC- and Havel-mito-like viruses

Hundreds of novel mitovirus-like sequences have been published in recent years (e.g., reference 50), and most of these viruses are fungal viruses that replicate in mitochondria. Here, we add three new virus sequences from candidate members of the genus *Unuamitovirus* (Supplementary Fig. S3). Another virus using translation code 4, Havel mito-like virus 1, clusters at the root of the *Mitoviridae* branch (Supplementary Fig. S3). For this virus, we failed to identify a related viral sequence with significant similarity (data not shown). However, because few mitoviruses lack internal UGA codons, we suggest that this virus has a non-fungal host. While some mito-like viruses that use the standard translation code are proven plant viruses [35, 36], a few have been associated with various hosts, e.g., Beihai narna-like virus 26 (source: tunicate [50]), Hubei narna-like virus 24 (source: diptera [50]), Melbourne fly narnavirus 1 (source: drosophila), Halley virus (source: penguin), Dwyer narna-like virus (source: rabbit), Gergich narna-like virus (source: rabbit), and eight mito-like viruses from the Teltow Canal and Havel River. All of these viruses cluster in a single clade that is independent of the four established *Mitoviridae* genera (Supplementary Fig. S3). Except for sequence data and the source of the sample, no biological information is available. Hence, the significance of this finding is unclear.

### TC- and Havel-narna-like viruses

Recently, Dinan et al. distinguished two clades of narna-like virus, which they named "alphanarnaviruses" and "betanarnaviruses" [37]. The "alphanarnavirus" group is proposed to be comprised of two species, "*Narnavirus saccharomajor*" and "*Narnavirus saccharominor*", as well as a number of unclassified viruses, including five viruses from the Havel River and Teltow Canal. As the members of this clade have been detected in fungal and non-fungal hosts, it is unclear whether the narna-like viruses of the Teltow Canal and Havel River are mycoviruses. Interestingly, a few "alphanarnaviruses" have an aligned, uninterrupted complementary reverse-frame ORF (ambigrammatic sequence) [37–40]. This interesting feature requires further investigation, as it is still unclear whether the encoded protein is really expressed or has a function, but the avoidance of CUA, UUA, and UCA codons, which correspond to translation termination codons in the complementary strand, suggest that the rORF encodes a protein [37, 38]. Narna-like viruses with an rORF have been named "mycoambinarnaviruses" [40], but the phylogenetic analyses of Dinan et al. [37], DiRisi et al. [39], and our study (Supplementary Fig. S3) do not indicate that these viruses belong to a single monophyletic clade.

### TC-Zyba-like amalgaviruses

Both TC-zyba-like viruses exhibit the characteristic zybavirus gene layout with fusion of ORFs 1 and 2 by a programmed frameshift and cluster with zyba-like viruses collected from various sources (Fig. 3). TC-zyba-like virus 1 groups closely with Antonospora locustae virus 1 and Tetrodontophora bielanensis associated virus 1, which were recently suggested on the basis of sequence identity scores to comprise a new genus of the family *Amalgaviridae* [51]. Other viruses of this clade were associated with unspecified diptera (KX884149 [50]), beetles (MZ209909), mosquitoes (OL700164), and insectivorous bats (OR871395). The oomycete *Antonospora locustae* is a pathogen of grasshoppers, and it seems plausible that the remaining zyba-like viruses of this clade also infect oomycetes, which are pathogenic to insects. Even the bat-associated zyba-like virus might have found its way into the *Rhinolophus* intestine via a dietary route rather than infecting bats. Another clade of zyba-like virus sequences includes a yeast-infecting virus of the species *Zybavirus bailii*, two viruses of plant-infecting fungi (MZ079606, MZ079609), and viruses obtained from water (MW784004) and soil (MN034945) samples. Conidiobolus rugosus virus 1 (MZ600508) was obtained from a soil sample has been associated with the fungus *Capillidium rugosum* (*Conidiobolus rugosus*). Chen et al. have associated their Xisha Islands zybavirus (MW784004) from an environmental water sample with putative plant/fungal/bacterial hosts [48]. TC-zyba-like virus 2 from a Teltow Canal water sample matches this correlation.

### TC-Sclerophthora macrospora B-like virus

This virus shows similarity to oomycete-infecting SmBV but also to the Quinjiang sobemo-like viruses 4, 5, 28 (accession nos. PQ055480, PQ055481, and PQ055506) from crabs and a virus sequence from grassland soil (MN033517) (Figs. 4 and 5). The availability of only partial sequences, sparse information on host range, and a lack of virus isolates, however, hamper further classification attempts.

Overall, the great divergence of several botourmia-, *Ghabrivirales*-, mito-, and narna-like viruses from the Teltow Canal and Havel River reflects the fast-growing numbers of new, unassigned mycoviruses and may require the creation of further taxa to integrate them in the present virus taxonomy. Undoubtedly, further sampling will uncover more viruses related to those presented here and contribute to a coherent view of riverine viromes.

## Supplementary Information

Below is the link to the electronic supplementary material.

Supplementary file1 (PDF 1678 KB)

## Data Availability

The datasets used and analyzed in the current study are available at NCBI: BioProject: PRJNA1174387; Biosamples: SAMN44339963, SAMN44339964; Short Read Archive: SRR31035838, SRR31035839; GenBank accession numbers: PX098516-PX098770.

## References

[1] Ghabrial SA, Suzuki N (2009) Viruses of plant pathogenic fungi. Annu Rev Phytopathol 47:353–384. 10.1146/annurev-phyto-080508-081932 19400634 10.1146/annurev-phyto-080508-081932

[2] Parratt SR, Laine AL (2016) The role of hyperparasitism in microbial pathogen ecology and evolution. ISME J 10:1815–1822. 10.1038/ismej.2015.247 26784356 10.1038/ismej.2015.247 PMC5029149

[3] Liu S, Xie J, Cheng J, Li B, Chen T, Fu Y, Li G, Wang M, Jin H, Wan H, Jiang D (2016) Fungal DNA virus infects a mycophagous insect and utilizes it as a transmission vector. Proc Natl Acad Sci USA 113:12803–12808. 10.1073/pnas.1608013113 27791095 10.1073/pnas.1608013113 PMC5111676

[4] Dai R, Yang S, Pang T, Tian M, Wang H, Zhang D, Wu Y, Kondo H, Andika IB, Kang Z, Sun L (2024) Identification of a negative-strand RNA virus with natural plant and fungal host. Proc Natl Acad Sci USA 121:e2319582121. 10.1073/pnas.2319582121 38483998 10.1073/pnas.2319582121 PMC10962957

[5] Jia J, Jiang D, Xie J (2024) Viruses shuttle between fungi and plants. Trends Microbiol 32:620–621. 10.1016/j.tim.2024.04.01 38719702 10.1016/j.tim.2024.04.013

[6] Andika IB, Wei S, Cao C, Salaipeth L, Kondo H, Sun L (2017) Phytopathogenic fungus hosts a plant virus: a naturally occurring cross-kingdom viral infection. Proc Natl Acad Sci USA 114:12267–12272. 10.1073/pnas.1714916114 29087346 10.1073/pnas.1714916114 PMC5699089

[7] Sutela S, Poimala A, Vainio EJ (2019) Viruses of fungi and oomycetes in the soil environment. FEMS Microbiol Ecol 95:fiz119. 10.1093/femsec/fiz119 31365065 10.1093/femsec/fiz119

[8] Grossart HP, Van den Wyngaert S, Kagami M, Wurzbacher C, Cunliffe M, Rojas-Jimenez K (2019) Fungi in aquatic systems. Nat Rev Microbiol 17:340–354. 10.1038/s41579-019-0175-8 10.1038/s41579-019-0175-8 30872817

[9] Ghabrial SA, Caston JR, Jiang D, Nibert M, Suzuki N (2015) 50-plus years of fungal viruses. Virology 479–480:356–368. 10.1016/j.virol.2015.02.034 25771805 10.1016/j.virol.2015.02.034

[10] Kondo H, Botella L, Suzuki N (2022) Mycovirus diversity and evolution revealed/inferred from recent studies. Annu Rev Phytopathol 60:307–336. 10.1146/annurev-phyto-021621-122122 35609970 10.1146/annurev-phyto-021621-122122

[11] Dawe VH, Kuhn CW (1983) Virus-like particles in the aquatic fungus, Rhizidiomyces. Virology 130:10–20. 10.1016/0042-6822(83)90113-7 18639135 10.1016/0042-6822(83)90113-7

[12] Ayllón MA, Vainio EJ (2023) Mycoviruses as a part of the global virome: Diversity, evolutionary links and lifestyle. Adv Virus Res 115:1–86. 10.1016/bx.aivir.2023.02.002 37173063 10.1016/bs.aivir.2023.02.002

[13] Sato Y, Suzuki N (2023) Continued mycovirus discovery expanding our understanding of virus lifestyles, symptom expression, and host defense. Curr Opin Microbiol 75:102337. 10.1016/j.mib.2023.102337 37343415 10.1016/j.mib.2023.102337

[14] Abdoulaye AH, Foda MF, Kotta-Loizou I (2019) Viruses infecting the plant pathogenic fungus *Rhizoctonia solani*. Viruses 11:1113. 10.3390/v11121113 31801308 10.3390/v11121113 PMC6950361

[15] Forgia M, Daghino S, Chiapello M, Ciuffo M, Turina M (2024) New clades of viruses infecting the obligatory biotroph *Bremia lactucae* representing distinct evolutionary trjectory for viruses infecting oomycetes. Virus Evol 10:1–16. 10.1093/ve/veae003 10.1093/ve/veae003 PMC10868552 38361818

[16] Andika IB, Tian M, Bian R, Cao X, Luo M, Kondo H, Sun L (2023) Cross-Kingdom interactions between plant and fungal viruses. Annu Rev Virol 10:119–138. 10.1046/annurev-virology-111821-122539 37406341 10.1146/annurev-virology-111821-122539

[17] Zell R, Groth M, Selinka L, Selinka HC (2022) Picorna-like viruses of the Havel River. Germany. Front Microbiol 13:865287. 10.3389/fmicb.2022.865287 35444619 10.3389/fmicb.2022.865287 PMC9013969

[18] Zell R, Groth M, Selinka L, Selinka HC (2023) Hepeliviruses in two waterbodies in Berlin. Germany. Arch Virol 168:9. 10.1007/s00705-022-05688-0 10.1007/s00705-022-05688-0 PMC9790848 36566475

[19] Zell R, Groth M, Selinka L, Selinka HC (2023) Exploring the diversity of plant-associated viruses and related viruses in riverine freshwater samples collected in Berlin, Germany. Pathogens 12:1458. 10.3390/pathogens12121458 38133341 10.3390/pathogens12121458 PMC10745976

[20] Zell R, Groth M, Selinka L, Selinka HC (2024) Diversity of picorna-like viruses in the Teltow Canal, Berlin. Germany. Viruses 16:1020. 10.3390/v16071020 39066183 10.3390/v16071020 PMC11281612

[21] Zell R, Groth M, Selinka L, Selinka HC (2024) Metagenomic analyses of water samples of two urban freshwaters in Berlin, Germany, reveal new highly diverse invertebrate viruses. Microorganisms 12:2361. 10.3390/microorganisms12112361 39597750 10.3390/microorganisms12112361 PMC11596407

[22] Wyn-Jones AP, Carducci A, Cook N, D'gostino MD, Divizia M, Fleischer J, Gantzer A, Girones R, Höller C, de Roda Husman AM, Kay D, Kozyra I, López-Pila J, Muscillo M, Sao José Nascimento M, Papgeorgiou G, Rutjes S, Sellwood J, Szewzyk R, Wyer M (2011) Surveillance of adenoviruses and noroviruses in European recreational waters. Water Res 45:1025−1038. 10.1016/j.waterres.2010.10.015 10.1016/j.watres.2010.10.015 PMC7112131 21093010

[23] Martin M (2011) Cutadapt removes adapter sequences from high-throughput sequence reads. EMBnet J 17:10–12. 10.14806/ej.17.1.200

[24] Nurk S, Melshko D, Korobeynikov A, Pevzner PA (2017) metaSPAdes: A new versatile metagenomic assembler. Genome Res 27:824–834. 10.1101/gr.213959.116 28298430 10.1101/gr.213959.116 PMC5411777

[25] Buchfink B, Xie C, Huson DH (2015) Fast and sensitive protein alignment using DIAMOND. Nat Methods 12:59–60. 10.1038/nmeth.3176 25402007 10.1038/nmeth.3176

[26] Kumar S, Stecher G, Li M, Knyaz C, Tamura K (2018) Molecular evolutionary genetics analysis across computing platforms. Mol Biol Evol 35:1547–1549. 10.1093/molbev/msy096 29722887 10.1093/molbev/msy096 PMC5967553

[27] Nguyen LT, Schmidt HA, von Haeseler A, Minh BQ (2015) IQ-TREE: A fast and effective stochastic algorithm for estimating maximum likelihood phylogenies. Mol Biol Evol 32:268–274. 10.1093/molbev/msu300 25371430 10.1093/molbev/msu300 PMC4271533

[28] Hoang DT, Chernomor O, von Haeseler A, Minh BQ (2018) UFBoot2: Improving the ultrafast bootstrap approximation. Mol Biol Evol 35:518–522. 10.1093/molbev/msx281 29077904 10.1093/molbev/msx281 PMC5850222

[29] Donaire L, Xie J, Nerva L, Jiang D, Marzano SYL, Sabanadzovic S, Turina M, Ayllon MA (2024) ICTV virus taxonomy profile: *Botourmiaviridae* 2024. J Gen Virol 105:002047. 10.1099/jgv.0.002047 39570657 10.1099/jgv.0.002047 PMC12453402

[30] Wolf YI, Kazlauskas D, Iranzo J, Lucía-Sanz A, Kuhn JH, Krupovic M, Dolja VV, Koonin EV (2018) Origins and evolution of the global RNA virome. mBio 9:e02329-18. 10.1128/mBio.02329-18 10.1128/mBio.02329-18 PMC6282212 30482837

[31] Koonin EV, Dolja VV, Krupovic M, Varsani A, Wolf YI, Yutin N, Zerbini M, Kuhn JH (2019) Proposal 2019.006G.a.v1.Riboviria. Create a metataxonomic framework, filling all principal taxonomic ranks, for realm *Riboviria*. https://talk.ictvonline.org/ictv/proposals/2019.006G.zip. Accessed 25 June 2025

[32] Naitow H, Tang J, Canady M, Wickner RB, Johnson JE (2002) L-A virus at 3.4 Å resolution reveals particle architecture and mRNA decapping mechanism. Nat Struct Biol 9:725–728. 10.1038/nsb844 12244300 10.1038/nsb844

[33] Botella L, Manny AR, Nibert ML, Vainio E (2021) Create 100 new species and four new genera (*Cryppavirales*: *Mitoviridae*). https://talk.ictvonline.org/ictv/proposals/2021.003F.R.Mitoviridae_100nsp_4ngen.zip. Accessed 25 June 2025

[34] Nibert M (2017) Mitovirus UGA(Trp) codon usage parallels that of host mitochondria. Virology 507:96–100. 10.1016/j.virol.2017.04.010 28431284 10.1016/j.virol.2017.04.010 PMC5517309

[35] Nibert ML, Vong M, Fugate KK, Debat HJ (2018) Evidence for contemporary plant mitoviruses. Virology 518:14–24. 10.1016/j.virol.2018.02.005 29438872 10.1016/j.virol.2018.02.005 PMC6668999

[36] Nerva L, Vigani G, Di Silvestre D, Ciuffo M, Forgia M, Chitarra W, Turina M (2019) Biological and molecular characterization of Chenopodium quinoa mitovirus 1 reveals a distinct small RNA response compared to those of cytoplasmic RNA viruses. J Virol 93:e01998-e2018. 10.1128/JVI.01998-18 30651361 10.1128/JVI.01998-18 PMC6430534

[37] Dinan AM, Lukhovitskaya NI, Olendraite I, Firth AE (2020) A case for a negative-strand coding sequence in a group of positive-sense RNA viruses. Virus Evol 6:veaa007. 10.1093/ve/veaa007 10.1093/ve/veaa007 PMC7010960 32064120

[38] Cook S, Chung BYW, Bass D, Moureau G, Tang S, McAlister E, Culverwell CL, Glücksman E, Wang H, Brown TDK, Gould EA, Harbach RE, de Lamballerie X, Firth AE (2013) Novel virus discovery and genome reconstruction from field RNA samples reveals highly divergent viruses in dipteran hosts. PLoS ONE 8:e80720. 10.1371/journal.pone.0080720 24260463 10.1371/journal.pone.0080720 PMC3832450

[39] DiRisi JL, Huber G, Kistler A, Retallack H, Wilkinson M, Yllanes D (2019) An exploration of ambigrammatic sequences in narnaviruses. Sci Rep 9:17982. 10.1038/s41598-019-54181-3 31784609 10.1038/s41598-019-54181-3 PMC6884476

[40] Chiapello M, Rodríguez-Romero J, Ayllón MA, Turina M (2020) Analysis of the virome associated to grapevine downy mildew lesions reveals new mycovirus lineages. Virus Evol 6:veaa058. 10.1093/ve/veaa058 10.1093/ve/veaa058 PMC7724247 33324489

[41] Depierreux D, Vong M, Nibert ML (2016) Nucleotide sequence of Zygosaccharomyces bailii virus Z: Evidence for +1 programmed ribosomal frameshifting and for assignment to family *Amalgaviridae*. Virus Res 217:115–124. 10.1016/j.virusres.2016.02.008 26951859 10.1016/j.virusres.2016.02.008 PMC5517306

[42] Honkura R, Shrako Y, Ehara Y, Yamanaka S (1983) Two types of virus-like particles isolated from downy mildew diseased rice plants. Ann Phytopathol Soc Jpn 49:653–658. 10.3186/jjphytopath.49.653

[43] Shirako Y, Ehara Y (1985) Composition of viruses isolated from Sclerophthora macrospora-infected rice plants. Ann Phytopath Soc Jpn 51:459–464. 10.3186/jjphytopath.51.459

[44] Heller-Dohmen M, Göpfert JC, Hammerschmidt R, Spring O (2008) Different pathotypes of the sunflower downy mildew pathogen *Plasmopara halstedii* all contain isometiric virions. Mol Plant Pathol 9:777–786. 10.1111/j.1364-3703.2008.00499.x 19019006 10.1111/j.1364-3703.2008.00499.x PMC6640286

[45] Heller-Dohmen M, Göpfert JC, Pfannstiel J, Spring O (2011) The nucleotide sequence and genome organization of *Plasmopara halstedii* virus. Virology J 8:123. 10.1186/1743-422X-8-123 21410989 10.1186/1743-422X-8-123 PMC3069955

[46] Yokoi T, Yamashita S, Hibi T (2003) The nucleotide sequence and genome organization of *Sclerophthora macrospora* virus A. Virology 311:394–399. 10.1016/S0042-6822(03)00183-1 12842628 10.1016/s0042-6822(03)00183-1

[47] Yokoi T, Takemoto Y, Suzuki M, Yamashita S, Hibi T (1999) The nucleotide sequence and genome organization of *Sclerophthora macrospora* virus B. Virology 264:344–349. 10.1006/viro.1999.0018 10562496 10.1006/viro.1999.0018

[48] Chen YM, Sadiq S, Tian JH, Chen X, Lin XD, Shen JJ, Chen H, Hao ZY, Wille M, Zhou ZC, Wu J, Li F, Wang HW, Yang WD, Xu QY, Wang W, Gao WH, Holmes EC, Zhang YZ (2022) RNA viromes from terrestrial sites across China expand environmental viral diversity. Nat Microbiol 7:1312–1323. 10.1038/s41564-022-01180-2 35902778 10.1038/s41564-022-01180-2

[49] Sadiq S, Harvey E, Mifsud JCO, Minasny B, McBratney AB, Pozza LE, Mahar JE, Holmes EC (2024) Australian terrestrial environments harbour extensive RNA virus diversity. Virology 593:110007. 10.1016/j.virol.2024.110007 38346363 10.1016/j.virol.2024.110007

[50] Shi M, Lin XD, Tian JH, Chen LJ, Chen X, Li CX, Qin XC, Li J, Cao JP, Eden JS, Buchmann J, Wang W, Xu J, Holmes EC, Zhang YZ (2016) Redefining the invertebrate RNA viroshere. Nature 540:539–543. 10.1038/nature20167 27880757 10.1038/nature20167

[51] Pyle JD, Keeling PJ, Nibert ML (2017) Amalga-like virus infecting Antonospora locustae, a microsporidian pathogen of grasshoppers, plus related viruses associated with other arthropods. Virus Res 233:95–104. 10.1016/j.virusres.2017.02.015 28267607 10.1016/j.virusres.2017.02.015 PMC5489755

