# Supplementary material for: A metagenomic analysis of urban river samples reveals high numbers of sequences related to mycoviruses
Source: Arch Virol. 2026 Jan 8;171(2):46. doi: 10.1007/s00705-025-06496-y (PMC12779748; doi:10.1007/s00705-025-06496-y)
Supplement: Supplementary file 1 — Supplementary file1 (PDF 1678 KB) [file 705_2025_6496_MOESM1_ESM.pdf]

Legends to Supplementary Figures

Supplementary Figure 1: Phylogenetic analysis of 260 RdRP sequences of members of the *Botourmiaviridae*, *Mitoviridae*, *Narnaviridae* and related viruses. The representative replicase sequences of members of all genera of the *Botourmiaviridae,* and unclassified related viruses from Teltow Canal, Havel River and various hosts as well as *Mitoviridae* and *Narnaviridae* (as outgroup) were aligned with MEGA and used for maximum likelihood tree inference with IQ-TREE 2 (optimal substitution model: PMB+F+R9). Presented are GenBank acc. nos., species names (printed in bold and italics), vernacular names and strain designations if available (in round brackets). Solid square brackets present names of genera (thin lines) and families (thick lines). Broken square brackets indicate proposed "alpha-" and "betanarnaviruses". Numbers at nodes indicate bootstrap support greater 65% obtained after 10,000 ultrafast replications. The bar indicates amino acid substitutions per site. Color code: black, classified viruses; blue, unclassified viruses; red, viruses from Teltow Canal and Havel River. A filled triangle (▲) indicates viruses with a dicistronic gene layout.

Supplementary Figure 2: Phylogenetic analysis of 142 RdRP sequences of members of the *Ghabrivirales* and related viruses. The representative replicase sequences of members of all families of the *Ghabrivirales* order and unclassified related viruses from Teltow Canal, Havel River and various hosts were aligned with MEGA and used for maximum likelihood tree inference with IQ-TREE 2 (optimal substitution model: VT+F+R9). Presented are GenBank acc. nos., species names (printed in bold and italics), vernacular names and strain designations if available (in round brackets). Square brackets indicate higher order taxa (family, suborder, order). The yellow box highlights sequences with internal termination codons in any tested translation table. Numbers at nodes indicate bootstrap support obtained after 10,000 ultrafast replications. The tree was arbitrarily rooted with the sequence of Fusarium graminearum alternavirus 1 (*Alternaviridae*, *Gammatotivirineae*). The bar indicates amino acid substitutions per site. Color code: black, classified viruses; blue, unclassified viruses; red, viruses from Teltow Canal and Havel River.

Supplementary Figure 3: Phylogenetic analysis of 153 RdRP sequences of members of the *Mitoviridae*, *Narnaviridae* and related viruses. The representative replicase sequences of members of all species of the *Mitoviridae* and *Narnaviridae*, and unclassified related viruses from Teltow Canal, Havel River and various hosts were aligned with MEGA and used for maximum likelihood tree inference with IQ-TREE 2 (optimal substitution model: PMB+F+R9). Presented are GenBank acc. nos., species names (printed in bold and italics), vernacular names and strain designations if available (in round brackets). Solid square brackets present genera (thin lines) and families (thick lines). Broken square brackets indicate proposed "alpha-" and "betanarnaviruses". Yellow boxes highlight viruses which use the standard translation table. Numbers at nodes indicate bootstrap support greater 65% obtained after 10,000 ultrafast replications. The tree was arbitrarily rooted with narnavirus sequences. The bar indicates amino acid substitutions per site. Color code: black, classified viruses; blue, unclassified viruses; red, viruses from Teltow Canal and Havel River.

Supplementary Figure 1

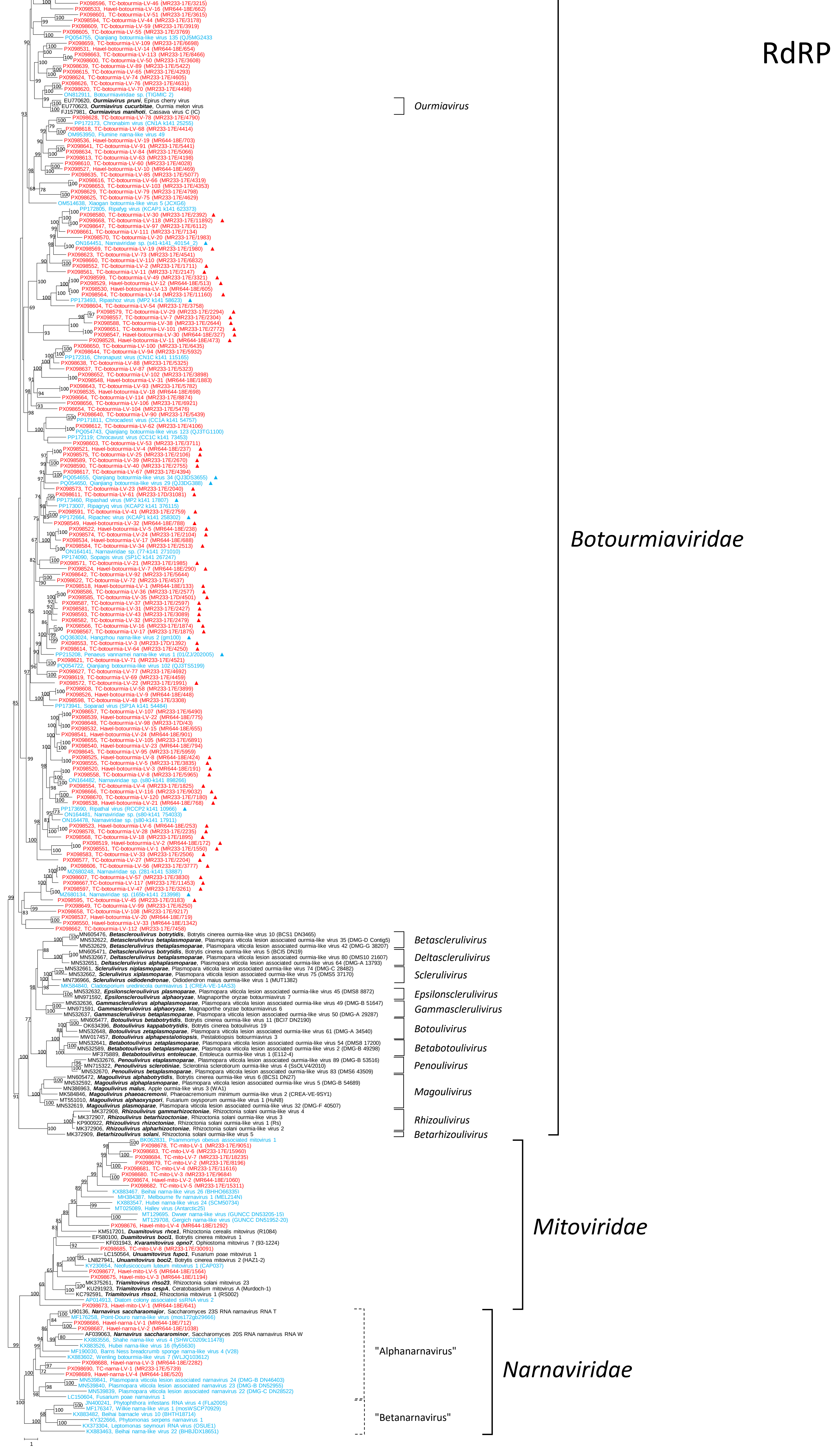

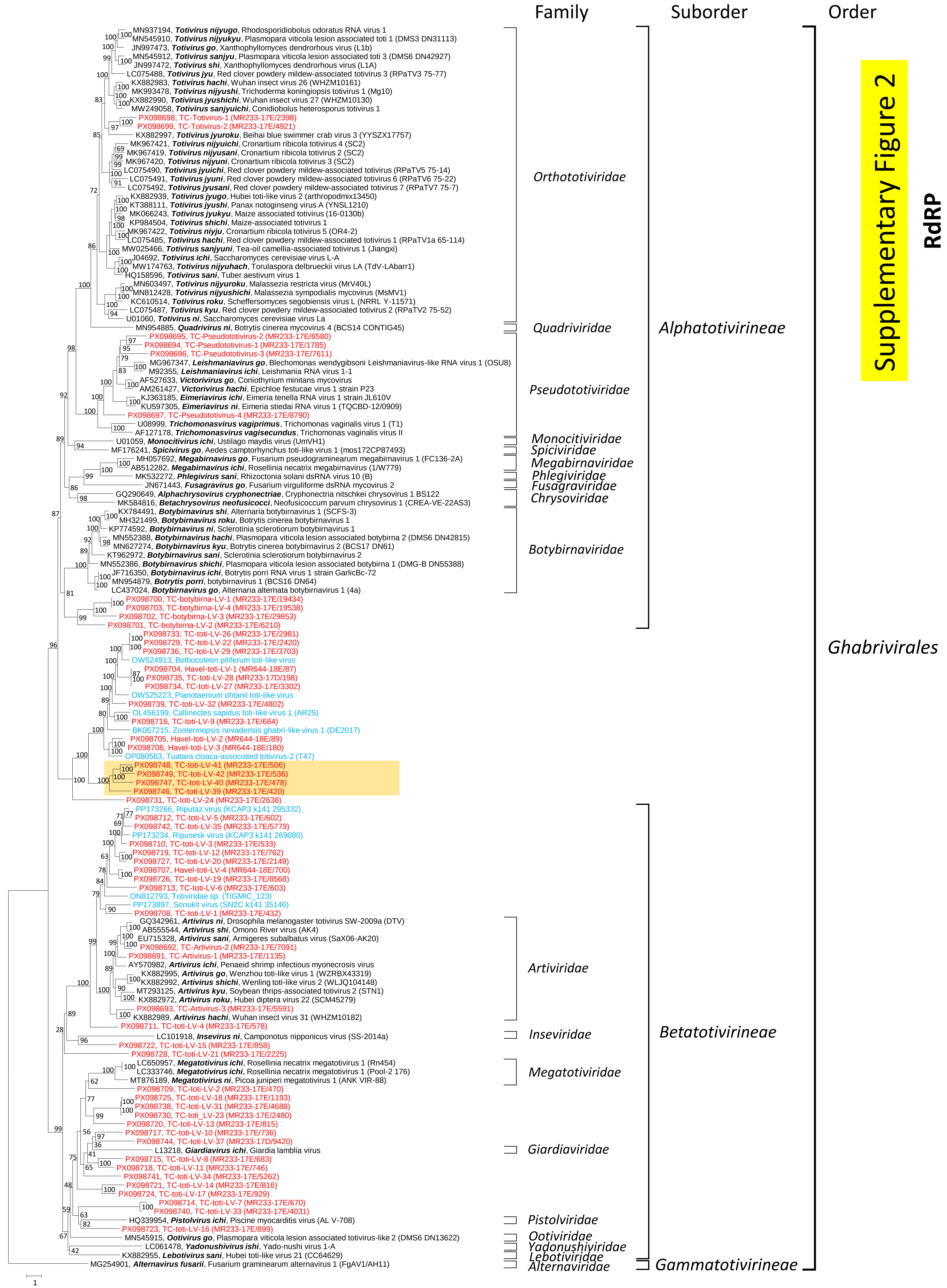

Supplementary Figure 2

Supplementary Figure 3

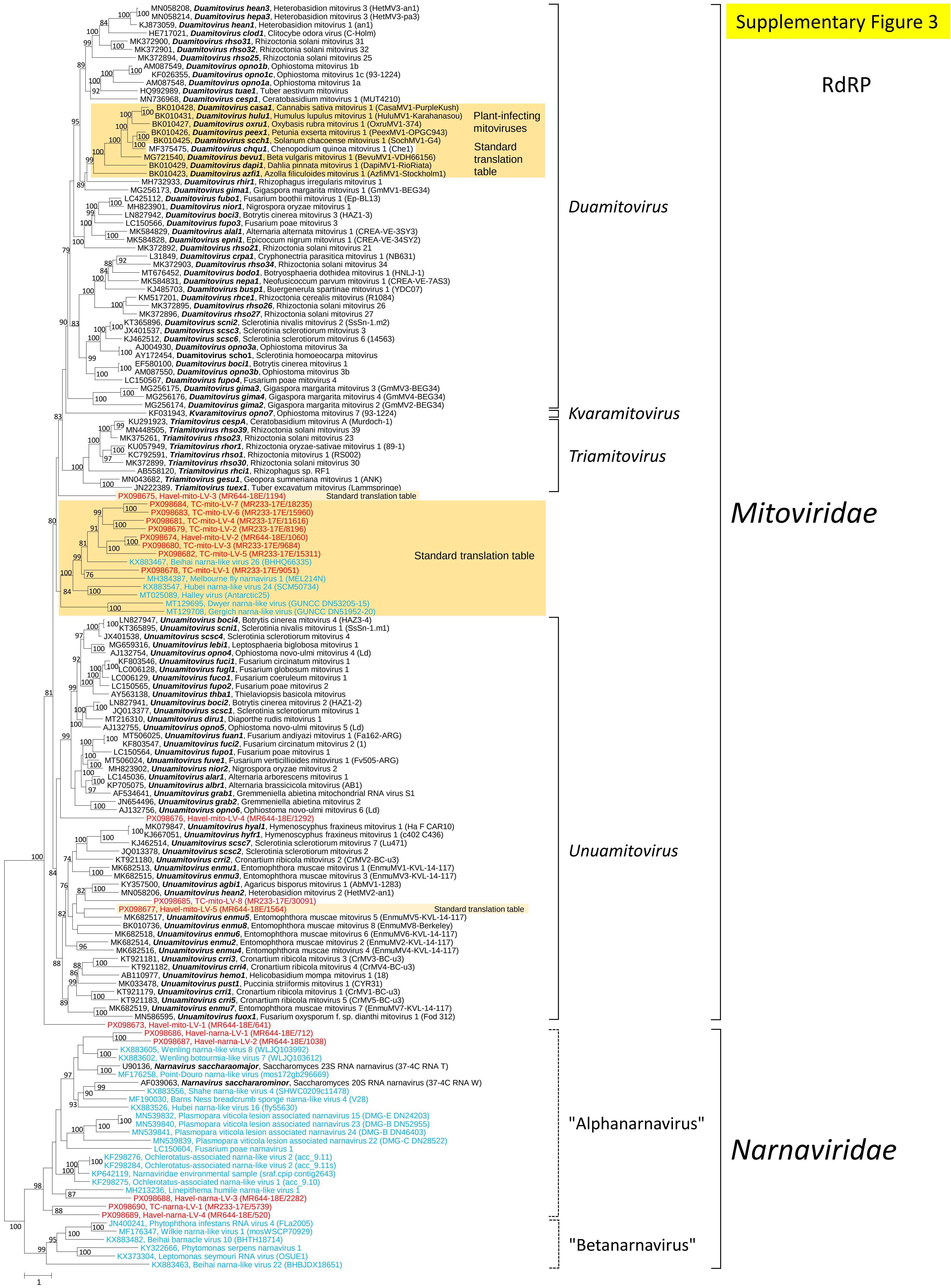

Seq #	GenBank acc.	Virus name and strain designation	Length	Completeness	Mean depth	Genome layout, conserved domains
seq1	PX098516	Teltow Canal zyba-like virus 1 MR233-17E/3953	3185 nt	partial	15.5755	**orf1-par:** hypothetical protein; **orf2-par:** fusion protein/polyprotein with RdRp domain (pfam00680, cd01699)
seq2	PX098517	Teltow Canal zyba-like virus 2 MR233-17E/18398	1433 nt	partial	56.9484	**orf-par:** RdRp domain (pfam00680, cd01699)
seq3	PX098518	Havel botourmia-like virus 1 MR644-18E/133	5664 nt	(almost) complete genome	61.2738	**orf1:** RdRp (cd23183); **orf2:** structural protein (pfam00729)
seq4	PX098519	Havel botourmia-like virus 2 MR644-18E/172	5048 nt	partial	240.865	**orf1-par:** RdRp (cd23183); **orf2:** structural protein (pfam00729)
seq5	PX098520	Havel botourmia-like virus 3 MR644-18E/191	4826 nt	(almost) complete genome	19.1337	**orf1:** RdRp (cd23183); **orf2:** structural protein (pfam00729)
seq6	PX098521	Havel botourmia-like virus 4 MR644-18E/237	4371 nt	partial	2221.67	**orf1-par:** RdRp (cd23183); **orf2:** structural protein (pfam00729)
seq7	PX098522	Havel botourmia-like virus 5 MR644-18E/238	4364 nt	partial	28.5406	**orf1-par:** RdRp (cd23183); **orf2:** structural protein (pfam00729)
seq8	PX098523	Havel botourmia-like virus 6 MR644-18E/253	4237 nt	partial	94.1808	**orf1-par:** RdRp (cd23183); **orf2:** structural protein (pfam00729)
seq9	PX098524	Havel botourmia-like virus 7 MR644-18E/290	3975 nt	(almost) complete genome	16.1769	**orf1:** RdRp (cd23183); **orf2:** structural protein (pfam00729)
seq10	PX098525	Havel botourmia-like virus 8 MR644-18E/424	3310 nt	(almost) complete genome	189.183	**orf1:** RdRp (cd23183); **orf2:** structural protein (pfam00729)
seq11	PX098526	Havel botourmia-like virus 9 MR644-18E/448	3176 nt	partial	18.7868	**orf-par:** RdRp (cd23183)
seq12	PX098527	Havel botourmia-like virus 10 MR644-18E/469	3083 nt	partial	248.118	**orf-par:** RdRp (cd23183)
seq13	PX098528	Havel botourmia-like virus 11 MR644-18E/473	3070 nt	partial	13.5958	**orf1-par:** RdRp (cd23183); **orf2:** structural protein (pfam00729)
seq14	PX098529	Havel botourmia-like virus 12 MR644-18E/513	4539 nt	partial	39.3503	**orf1-par:** RdRp (cd23183); **orf2:** structural protein (pfam00729)
seq15	PX098530	Havel botourmia-like virus 13 MR644-18E/605	2701 nt	partial	41.3084	**orf-par:** RdRp (cd23183)
seq16	PX098531	Havel botourmia-like virus 14 MR644-18E/654	2580 nt	partial	38.4384	**orf-par:** RdRp (cd23183)
seq17	PX098532	Havel botourmia-like virus 15 MR644-18E/655	2524 nt	partial	35.4354	**orf:** RdRp (cd23183)
seq18	PX098533	Havel botourmia-like virus 16 MR644-18E/662	2572 nt	partial	11.5365	**orf-par:** RdRp (cd23183)
seq19	PX098534	Havel botourmia-like virus 17 MR644-18E/688	2511 nt	partial	16.3664	**orf-par:** RdRp (cd23183)
seq20	PX098535	Havel botourmia-like virus 18 MR644-18E/698	2494 nt	partial	34.8998	**orf-par:** RdRp (cd23183)
seq21	PX098536	Havel botourmia-like virus 19 MR644-18E/703	2988 nt	partial	7.39692	**orf-par:** RdRp (cd23183)
seq22	PX098537	Havel botourmia-like virus 20 MR644-18E/719	2463 nt	partial	16.0601	**orf-par:** RdRp (cd23183)
seq23	PX098538	Havel botourmia-like virus 21 MR644-18E/768	3809 nt	partial	26.586	**orf1-par:** RdRp (cd23183); **orf2:** structural protein (pfam00729)
seq24	PX098539	Havel botourmia-like virus 22 MR644-18E/775	2377 nt	partial	824.501	**orf:** RdRp (cd23183)
seq25	PX098540	Havel botourmia-like virus 23 MR644-18E/794	2361 nt	partial	78.8759	**orf-par:** RdRp (cd23183)
seq26	PX098541	Havel botourmia-like virus 24 MR644-18E/901	2220 nt	partial	10.9419	**orf:** RdRp (cd23183)
seq27	PX098542	Havel botourmia-like virus 25 MR644-18E/1324	2787 nt	partial	20.4578	**orf:** RdRp (cd23183)
seq28	PX098543	Havel botourmia-like virus 26 MR644-18E/1338	1831 nt	partial	12.7794	**orf-par:** RdRp (cd23183)
seq29	PX098544	Havel botourmia-like virus 27 MR644-18E/1650	1656 nt	partial	9.54408	**orf-par:** RdRp (cd23183)
seq30	PX098545	Havel botourmia-like virus 28 MR644-18E/1696	1639 nt	partial	6.44478	**orf-par:** RdRp (cd23183)
seq31	PX098546	Havel botourmia-like virus 29 MR644-18E/1911	1559 nt	partial	15.9705	**orf-par:** RdRp (cd23183)
seq32	PX098547	Havel botourmia-like virus 30 MR644-18E/327	3745 nt	partial	19.7274	**orf1-par:** RdRp (cd23183); **orf2:** structural protein (pfam00729)
seq33	PX098548	Havel botourmia-like virus 31 MR644-18E/1883	2282 nt	partial	11.9947	**orf-par:** RdRp (cd23183)
seq34	PX098549	Havel botourmia-like virus 32 MR644-18D/50637	2747 nt	partial	6.29924	**orf1-par:** RdRp (cd23183); **orf2-par:** structural protein (pfam00729)
seq35	PX098550	Havel botourmia-like virus 33 MR644-18E/1342	1828 nt	partial	33.4666	**orf-par:** RdRp (cd23183)
seq36	PX098551	Teltow Canal botourmia-like virus 1 MR233-17E/1550	5081 nt	partial	712.5	**orf1-par:** RdRp (cd23183); **orf2:** structural protein (pfam00729)
seq37	PX098552	Teltow Canal botourmia-like virus 2 MR233-17E/1711	4853 nt	(almost) complete genome	104.097	**orf1:** RdRp (cd23183); **orf2:** structural protein (pfam00729)
seq38	PX098553	Teltow Canal botourmia-like virus 3 MR233-17D/1392	4770 nt	(almost) complete genome	2180.25	**orf1:** RdRp (cd23183); **orf2:** structural protein (pfam00729)
seq39	PX098554	Teltow Canal botourmia-like virus 4 MR233-17E/1825	4685 nt	(almost) complete genome	453.612	**orf1:** RdRp (cd23183); **orf2:** structural protein (pfam00729)
seq40	PX098555	Teltow Canal botourmia-like virus 5 MR233-17E/3835	3313 nt	(almost) complete genome	1676.87	**orf1:** RdRp (cd23183); **orf2:** structural protein (pfam00729)
seq41	PX098556	Teltow Canal botourmia-like virus 6 MR233-17E/11717	1800 nt	partial	28.785	**orf1-par:** hypothetical protein; **orf2:** structural protein (pfam00729)
seq42	PX098557	Teltow Canal botourmia-like virus 7 MR233-17E/2304	4158 nt	partial	586.521	**orf1-par:** RdRp (cd23183); **orf2:** structural protein (pfam00729)
seq43	PX098558	Teltow Canal botourmia-like virus 8 MR233-17E/5965	4798 nt	(almost) complete genome	25.5932	**orf1:** RdRp (cd23183); **orf2:** structural protein (pfam00729)
seq44	PX098559	Teltow Canal botourmia-like virus 9 MR233-17E/17530	1469 nt	partial	6.85432	**orf1-par:** hypothetical protein; **orf2:** structural protein (pfam00729)
seq45	PX098560	Teltow Canal botourmia-like virus 10 MR233-17E/25852	2326 nt	partial	18797.3	**orf1-par:** RdRp (cd23183); **orf2:** structural protein (pfam00729)
seq46	PX098561	Teltow Canal botourmia-like virus 11 MR233-17E/2147	4314 nt	partial	25.2543	**orf1-par:** RdRp (cd23183); **orf2-par:** structural protein (pfam00729)
seq47	PX098562	Teltow Canal botourmia-like virus 12 MR233-17E/9352	2016 nt	partial	7.43552	**orf-par:** RdRp (cd23183)
seq48	PX098563	Teltow Canal botourmia-like virus 13 MR233-17E/9309	2022 nt	partial	12.8511	**orf-par:** RdRp (cd23183)
seq49	PX098564	Teltow Canal botourmia-like virus 14 MR233-17E/11160	4026 nt	partial	20.1508	**orf1-par:** RdRp (cd23183); **orf2:** structural protein (pfam00729)
seq50	PX098565	Teltow Canal botourmia-like virus 15 MR233-17E/9272	2026 nt	partial	10.5099	**orf-par:** RdRp (cd23183)
seq51	PX098566	Teltow Canal botourmia-like virus 16 MR233-17E/1874	4623 nt	partial	547.434	**orf1-par:** RdRp (cd23183); **orf2:** structural protein (pfam00729)
seq52	PX098567	Teltow Canal botourmia-like virus 17 MR233-17E/1875	4621 nt	partial	47.1372	**orf1-par:** RdRp (cd23183); **orf2:** structural protein (pfam00729); **orf3:** hypothetical protein
seq53	PX098568	Teltow Canal botourmia-like virus 18 MR233-17E/1895	4603 nt	(almost) complete genome	60.8657	**orf1:** RdRp (cd23183); **orf2:** hypothetical protein
seq54	PX098569	Teltow Canal botourmia-like virus 19 MR233-17E/1980	4496 nt	partial	16.5905	**orf1-par:** RdRp (cd23183); **orf2:** structural protein (pfam00729)
seq55	PX098570	Teltow Canal botourmia-like virus 20 MR233-17E/1983	4491 nt	partial	49.2084	**orf1-par:** RdRp (cd23183); **orf2:** hypothetical protein
seq56	PX098571	Teltow Canal botourmia-like virus 21 MR233-17E/1985	4487 nt	partial	42.064	**orf1-par:** RdRp (cd23183); **orf2:** structural protein (pfam00729)
seq57	PX098572	Teltow Canal botourmia-like virus 22 MR233-17E/1991	4479 nt	partial	24.0493	**orf1-par:** RdRp (cd23183); **orf2:** hypothetical protein
seq58	PX098573	Teltow Canal botourmia-like virus 23 MR233-17E/2040	4428 nt	(almost) complete genome	100.733	**orf1:** RdRp (cd23183); **orf2:** structural protein (pfam00729)
seq59	PX098574	Teltow Canal botourmia-like virus 24 MR233-17E/2104	4364 nt	partial	1047.15	**orf1-par:** RdRp (cd23183); **orf2:** structural protein (pfam00729)
seq60	PX098575	Teltow Canal botourmia-like virus 25 MR233-17E/2106	4360 nt	partial	488.924	**orf1-par:** RdRp (cd23183); **orf2:** structural protein (pfam00729)
seq61	PX098576	Teltow Canal botourmia-like virus 26 MR233-17E/2162	4300 nt	partial	284.134	**orf:** RdRp (cd23183)
seq62	PX098577	Teltow Canal botourmia-like virus 27 MR233-17E/2204	4260 nt	partial	27.7366	**orf1-par:** RdRp (cd23183); **orf2:** hypothetical protein
seq63	PX098578	Teltow Canal botourmia-like virus 28 MR233-17E/2235	4228 nt	partial	95.039	**orf1-par:** RdRp (cd23183); **orf2:** structural protein (pfam00729)

seq64	PX098579	Teltow Canal botourmia-like virus 29 MR233-17E/2294	4170 nt	partial	61.7118	**orf1-par:** RdRp (cd23183); **orf2:** structural protein (pfam00729)
seq65	PX098580	Teltow Canal botourmia-like virus 30 MR233-17E/2392	4076 nt	partial	89.9156	**orf1-par:** RdRp (cd23183); **orf2:** structural protein (pfam00729)
seq66	PX098581	Teltow Canal botourmia-like virus 31 MR233-17E/2427	4049 nt	partial	970.132	**orf1-par:** RdRp (cd23183); **orf2:** structural protein (pfam00729)
seq67	PX098582	Teltow Canal botourmia-like virus 32 MR233-17E/2479	4011 nt	(almost) complete genome	150.966	**orf1:** RdRp (cd23183); **orf2:** structural protein (pfam00729)
seq68	PX098583	Teltow Canal botourmia-like virus 33 MR233-17E/2506	3987 nt	partial	1949.23	**orf1-par:** RdRp (cd23183); **orf2:** structural protein (pfam00729)
seq69	PX098584	Teltow Canal botourmia-like virus 34 MR233-17E/2513	3979 nt	partial	578.21	**orf1-par:** RdRp (cd23183); **orf2:** structural protein (pfam00729)
seq70	PX098585	Teltow Canal botourmia-like virus 35 MR233-17D/4501	3983 nt	partial	186.078	**orf1:** RdRp (cd23183); **orf2-par:** structural protein (pfam00729)
seq71	PX098586	Teltow Canal botourmia-like virus 36 MR233-17E/2577	3932 nt	partial	86.1554	**orf1-par:** RdRp (cd23183); **orf2:** structural protein (pfam00729)
seq72	PX098587	Teltow Canal botourmia-like virus 37 MR233-17E/2597	3917 nt	(almost) complete genome	268.871	**orf1:** RdRp (cd23183); **orf2:** structural protein (pfam00729)
seq73	PX098588	Teltow Canal botourmia-like virus 38 MR233-17E/2644	3883 nt	partial	11.206	**orf1-par:** RdRp (cd23183); **orf2:** structural protein (pfam00729)
seq74	PX098589	Teltow Canal botourmia-like virus 39 MR233-17E/2670	3867 nt	(almost) complete genome	167.304	**orf1:** RdRp (cd23183); **orf2:** structural protein (pfam00729)
seq75	PX098590	Teltow Canal botourmia-like virus 40 MR233-17E/2755	3814 nt	partial	39.9216	**orf1-par:** RdRp (cd23183); **orf2:** structural protein (pfam00729)
seq76	PX098591	Teltow Canal botourmia-like virus 41 MR233-17E/2759	3812 nt	partial	20.6306	**orf1-par:** RdRp (cd23183); **orf2:** structural protein (pfam00729)
seq77	PX098592	Teltow Canal botourmia-like virus 42 MR233-17E/2812	3773 nt	partial	119.715	**orf1:** RdRp (cd23183)
seq78	PX098593	Teltow Canal botourmia-like virus 43 MR233-17E/3089	3607 nt	partial	35.3108	**orf1-par:** RdRp (cd23183); **orf2-par:** structural protein (pfam00729)
seq79	PX098594	Teltow Canal botourmia-like virus 44 MR233-17E/3178	3557 nt	partial	1989.53	**orf1-par:** RdRp (cd23183)
seq80	PX098595	Teltow Canal botourmia-like virus 45 MR233-17E/3183	3555 nt	partial	10.2838	**orf1-par:** RdRp (cd23183); **orf2:** hypothetical protein
seq81	PX098596	Teltow Canal botourmia-like virus 46 MR233-17E/3215	3543 nt	partial	25.0099	**orf1:** RdRp (cd23183)
seq82	PX098597	Teltow Canal botourmia-like virus 47 MR233-17E/3261	3520 nt	partial	176.672	**orf1-par:** RdRp (cd23183); **orf2:** structural protein (pfam00729)
seq83	PX098598	Teltow Canal botourmia-like virus 48 MR233-17E/3308	3489 nt	partial	30.6879	**orf1:** RdRp (cd23183)
seq84	PX098599	Teltow Canal botourmia-like virus 49 MR233-17E/3321	4502 nt	partial	18.5269	**orf1-par:** RdRp (cd23183); **orf2:** structural protein (pfam00729)
seq85	PX098600	Teltow Canal botourmia-like virus 50 MR233-17E/3608	3337 nt	partial	27.9865	**orf1:** RdRp (cd23183)
seq86	PX098601	Teltow Canal botourmia-like virus 51 MR233-17E/3615	3334 nt	partial	38.1758	**orf1-par:** RdRp (cd23183)
seq87	PX098602	Teltow Canal botourmia-like virus 52 MR233-17E/3649	3322 nt	partial	6.02438	**orf1:** RdRp (cd23183)
seq88	PX098603	Teltow Canal botourmia-like virus 53 MR233-17E/3711	3297 nt	partial	24.7452	**orf1:** RdRp (cd23183)
seq89	PX098604	Teltow Canal botourmia-like virus 54 MR233-17E/3758	3278 nt	partial	19.9109	**orf1-par:** RdRp (cd23183)
seq90	PX098605	Teltow Canal botourmia-like virus 55 MR233-17E/3769	3272 nt	partial	15.272	**orf1-par:** RdRp (cd23183)
seq91	PX098606	Teltow Canal botourmia-like virus 56 MR233-17E/3777	3266 nt	partial	104.822	**orf1-par:** RdRp (cd23183); **orf2:** structural protein (pfam00729)
seq92	PX098607	Teltow Canal botourmia-like virus 57 MR233-17E/3830	3240 nt	partial	40.2605	**orf1-par:** RdRp (cd23183); **orf2:** structural protein (pfam00729)
seq93	PX098608	Teltow Canal botourmia-like virus 58 MR233-17E/3899	3209 nt	partial	242.723	**orf1-par:** RdRp (cd23183)
seq94	PX098609	Teltow Canal botourmia-like virus 59 MR233-17E/3919	3202 nt	partial	25.7033	**orf1-par:** RdRp (cd23183)
seq95	PX098610	Teltow Canal botourmia-like virus 60 MR233-17E/4028	3148 nt	partial	27.4549	**orf1-par:** RdRp (cd23183)
seq96	PX098611	Teltow Canal botourmia-like virus 61 MR233-17D/31081	3898 nt	partial	10.7383	**orf1-par:** RdRp (cd23183); **orf2:** structural protein (pfam00729)
seq97	PX098612	Teltow Canal botourmia-like virus 62 MR233-17E/4106	3120 nt	partial	57.7952	**orf1-par:** RdRp (cd23183)
seq98	PX098613	Teltow Canal botourmia-like virus 63 MR233-17E/4198	3080 nt	partial	42.1208	**orf1:** RdRp (cd23183)
seq99	PX098614	Teltow Canal botourmia-like virus 64 MR233-17E/4250	3060 nt	partial	13.3154	**orf1-par:** RdRp (cd23183); **orf2:** structural protein (pfam00729)
seq100	PX098615	Teltow Canal botourmia-like virus 65 MR233-17E/4293	3039 nt	partial	169.976	**orf1-par:** RdRp (cd23183)
seq101	PX098616	Teltow Canal botourmia-like virus 66 MR233-17E/4319	3026 nt	partial	60.8199	**orf1-par:** RdRp (cd23183)
seq102	PX098617	Teltow Canal botourmia-like virus 67 MR233-17E/4394	3000 nt	partial	16.9357	**orf1-par:** RdRp (cd23183)
seq103	PX098618	Teltow Canal botourmia-like virus 68 MR233-17E/4414	2992 nt	partial	35.1671	**orf1-par:** RdRp (cd23183)
seq104	PX098619	Teltow Canal botourmia-like virus 69 MR233-17E/4459	2975 nt	partial	66.5694	**orf1-par:** RdRp (cd23183)
seq105	PX098620	Teltow Canal botourmia-like virus 70 MR233-17E/4498	2962 nt	partial	88.4818	**orf1-par:** RdRp (cd23183)
seq106	PX098621	Teltow Canal botourmia-like virus 71 MR233-17E/4521	2953 nt	partial	35.3691	**orf1-par:** RdRp (cd23183)
seq107	PX098622	Teltow Canal botourmia-like virus 72 MR233-17E/4537	2944 nt	partial	20.284	**orf1:** RdRp (cd23183)
seq108	PX098623	Teltow Canal botourmia-like virus 73 MR233-17E/4541	2942 nt	partial	30.5809	**orf1:** RdRp (cd23183)
seq109	PX098624	Teltow Canal botourmia-like virus 74 MR233-17E/4605	2921 nt	partial	90.5635	**orf1-par:** RdRp (cd23183)
seq110	PX098625	Teltow Canal botourmia-like virus 75 MR233-17E/4629	2915 nt	partial	38.8727	**orf1-par:** RdRp (cd23183)
seq111	PX098626	Teltow Canal botourmia-like virus 76 MR233-17E/4631	2913 nt	partial	260.218	**orf1:** RdRp (cd23183)
seq112	PX098627	Teltow Canal botourmia-like virus 77 MR233-17E/4692	2890 nt	partial	75.6121	**orf1-par:** RdRp (cd23183)
seq113	PX098628	Teltow Canal botourmia-like virus 78 MR233-17E/4790	2856 nt	partial	43.9762	**orf1-par:** RdRp (cd23183)
seq114	PX098629	Teltow Canal botourmia-like virus 79 MR233-17E/4798	2854 nt	partial	3372.47	**orf1:** RdRp (cd23183)
seq115	PX098630	Teltow Canal botourmia-like virus 80 MR233-17E/4888	2830 nt	partial	439.149	**orf1-par:** RdRp (cd23183)
seq116	PX098631	Teltow Canal botourmia-like virus 81 MR233-17E/4935	2817 nt	partial	225.662	**orf1-par:** RdRp (cd23183)
seq117	PX098632	Teltow Canal botourmia-like virus 82 MR233-17E/5059	2781 nt	partial	136.624	**orf1-par:** RdRp (cd23183)
seq118	PX098633	Teltow Canal botourmia-like virus 83 MR233-17E/5063	2779 nt	partial	31.7643	**orf1-par:** RdRp (cd23183)
seq119	PX098634	Teltow Canal botourmia-like virus 84 MR233-17E/5066	2778 nt	partial	247.333	**orf1-par:** RdRp (cd23183)
seq120	PX098635	Teltow Canal botourmia-like virus 85 MR233-17E/5077	2776 nt	partial	25.9316	**orf1-par:** RdRp (cd23183)
seq121	PX098636	Teltow Canal botourmia-like virus 86 MR233-17E/5082	2775 nt	partial	77.5802	**orf1:** RdRp (cd23183)
seq122	PX098637	Teltow Canal botourmia-like virus 87 MR233-17E/5323	2714 nt	partial	13.4816	**orf1:** RdRp (cd23183)
seq123	PX098638	Teltow Canal botourmia-like virus 88 MR233-17E/5325	2713 nt	partial	43.6436	**orf1:** RdRp (cd23183)
seq124	PX098639	Teltow Canal botourmia-like virus 89 MR233-17E/5422	2690 nt	partial	50.877	**orf1-par:** RdRp (cd23183)
seq125	PX098640	Teltow Canal botourmia-like virus 90 MR233-17E/5439	2684 nt	partial	14.5768	**orf1-par:** RdRp (cd23183)
seq126	PX098641	Teltow Canal botourmia-like virus 91 MR233-17E/5441	2683 nt	partial	423.114	**orf1-par:** RdRp (cd23183)
seq127	PX098642	Teltow Canal botourmia-like virus 92 MR233-17E/5644	2629 nt	partial	12.1563	**orf1-par:** RdRp (cd23183)
seq128	PX098643	Teltow Canal botourmia-like virus 93 MR233-17E/5782	2594 nt	partial	378.885	**orf1-par:** RdRp (cd23183)
seq129	PX098644	Teltow Canal botourmia-like virus 94 MR233-17E/5932	2560 nt	partial	19.6938	**orf1-par:** RdRp (cd23183)
seq130	PX098645	Teltow Canal botourmia-like virus 95 MR233-17E/5959	2553 nt	partial	600.629	**orf1-par:** RdRp (cd23183)
seq131	PX098646	Teltow Canal botourmia-like virus 96 MR233-17E/5994	2545 nt	partial	9.62122	**orf1-par:** RdRp (cd23183); **orf2-par:** structural protein (pfam00729)
seq132	PX098647	Teltow Canal botourmia-like virus 97 MR233-17E/6112	4031 nt	partial	32.7187	**orf1-par:** RdRp (cd23183); **orf2:** structural protein (pfam00729)
seq133	PX098648	Teltow Canal botourmia-like virus 98 MR233-17D/43	2688 nt	partial	15405.6	**orf1:** RdRp (cd23183)
seq134	PX098649	Teltow Canal botourmia-like virus 99 MR233-17E/6250	2489 nt	partial	251.713	**orf1:** RdRp (cd23183)
seq135	PX098650	Teltow Canal botourmia-like virus 100 MR233-17E/6435	2453 nt	partial	9.72768	**orf1-par:** RdRp (cd23183)
seq136	PX098651	Teltow Canal botourmia-like virus 101 MR233-17E/2772	3801 nt	partial	15575.8	**orf1-par:** RdRp (cd23183); **orf2:** structural protein (pfam00729)
seq137	PX098652	Teltow Canal botourmia-like virus 102 MR233-17E/3898	3210 nt	partial	937.051	**orf1:** RdRp (cd23183)
seq138	PX098653	Teltow Canal botourmia-like virus 103 MR233-17E/4353	3012 nt	partial	22.9426	**orf1-par:** RdRp (cd23183)
seq139	PX098654	Teltow Canal botourmia-like virus 104 MR233-17E/5476	2675 nt	partial	14.9151	**orf1:** RdRp (cd23183)
seq140	PX098655	Teltow Canal botourmia-like virus 105 MR233-17E/6891	2479 nt	partial	6906.3	**orf1-par:** RdRp (cd23183)
seq141	PX098656	Teltow Canal botourmia-like virus 106 MR233-17E/6921	2357 nt	partial	14.1803	**orf1-par:** RdRp (cd23183)
seq142	PX098657	Teltow Canal botourmia-like virus 107 MR233-17E/6490	2443 nt	partial	16119.8	**orf1:** RdRp (cd23183)
seq143	PX098658	Teltow Canal botourmia-like virus 108 MR233-17E/9217	2032 nt	partial	7.00541	**orf1-par:** RdRp (cd23183)
seq144	PX098659	Teltow Canal botourmia-like virus 109 MR233-17E/6698	2397 nt	partial	16.8652	**orf1-par:** RdRp (cd23183)
seq145	PX098660	Teltow Canal botourmia-like virus 110 MR233-17E/6832	2375 nt	partial	13.1912	**orf1-par:** RdRp (cd23183)

seq146	PX098661	Teltow Canal botourmia-like virus 111 MR233-17E/7134	2317 nt	partial	136.018	**orf1-par:** RdRp (cd23183)
seq147	PX098662	Teltow Canal botourmia-like virus 112 MR233-17E/7458	2266 nt	partial	17.7868	**orf1:** RdRp (cd23183)
seq148	PX098663	Teltow Canal botourmia-like virus 113 MR233-17E/8466	3210 nt	partial	137.539	**orf1:** RdRp (cd23183)
seq149	PX098664	Teltow Canal botourmia-like virus 114 MR233-17E/8874	2369 nt	partial	23.3634	**orf1-par:** RdRp (cd23183)
seq150	PX098665	Teltow Canal botourmia-like virus 115 MR233-17E/8925	2063 nt	partial	15.2763	**orf1-par:** RdRp (cd23183)
seq151	PX098666	Teltow Canal botourmia-like virus 116 MR233-17E/9032	4033 nt	(almost) complete genome	77.065	**orf1:** RdRp (cd23183); **orf2:** structural protein (pfam00729)
seq152	PX098667	Teltow Canal botourmia-like virus 117 MR233-17E/11453	3396 nt	partial	181.585	**orf1-par:** RdRp (cd23183); **orf2:** structural protein (pfam00729)
seq153	PX098668	Teltow Canal botourmia-like virus 118 MR233-17E/11892	3536 nt	partial	34.8931	**orf1-par:** RdRp (cd23183); **orf2:** structural protein (pfam00729)
seq154	PX098669	Teltow Canal botourmia-like virus 119 MR233-17D/49655	2242 nt	partial	8.75691	**orf1-par:** hypothetical protein; **orf2:** structural protein (pfam00729)
seq155	PX098670	Teltow Canal botourmia-like virus 120 MR233-17E/7180	4355 nt	partial	90.8955	**orf1-par:** RdRp (cd23183); **orf2:** structural protein (pfam00729)
seq156	PX098671	Teltow Canal botourmia-like virus 121 MR233-17E/5810	2589 nt	partial	15.4438	**orf-par:** helicase (pfam00910) - **RdRp** (cd23183)
seq157	PX098672	Teltow Canal Riboviria sp. MR233-17E/19337	1183 nt	partial	41.1293	**orf-par:** structural protein (pfam00729)
seq158	PX098673	Havel mito-like virus 1 MR644-18E/641	2613 nt	complete coding sequence	32.3551	**orf:** RdRp (pfam05919); translation table 4
seq159	PX098674	Havel mito-like virus 2 MR644-18E/1060	2053 nt	complete coding sequence	30.1583	**orf:** RdRp (pfam05919); translation table 4
seq160	PX098675	Havel mito-like virus 3 MR644-18E/1194	1947 nt	partial	25.5475	**orf:** RdRp (pfam05919); translation table 4
seq161	PX098676	Havel mito-like virus 4 MR644-18E/1292	1866 nt	partial	24.5252	**orf:** RdRp (pfam05919); translation table 4
seq162	PX098677	Havel mito-like virus 5 MR644-18E/1564	1701 nt	partial	11.5726	**orf:** RdRp (pfam05919); translation table 4
seq163	PX098678	Teltow Canal mito-like virus 1 MR233-17E/9051	2048 nt	partial	25.5347	**orf:** RdRp (pfam05919); translation table 4
seq164	PX098679	Teltow Canal mito-like virus 2 MR233-17E/8196	2157 nt	partial	15.3245	**orf:** RdRp (pfam05919); translation table 4
seq165	PX098680	Teltow Canal mito-like virus 3 MR233-17E/9684	1981 nt	partial	13.947	**orf:** RdRp (pfam05919); translation table 4
seq166	PX098681	Teltow Canal mito-like virus 4 MR233-17E/11616	1807 nt	partial	13.1184	**orf:** RdRp (pfam05919); translation table 4
seq167	PX098682	Teltow Canal mito-like virus 5 MR233-17E/15311	1945 nt	complete coding sequence	10.181	**orf:** RdRp (pfam05919); translation table 4
seq168	PX098683	Teltow Canal mito-like virus 6 MR233-17E/15960	1694 nt	partial	26.6741	**orf:** RdRp (pfam05919); translation table 4
seq169	PX098684	Teltow Canal mito-like virus 7 MR233-17E/18235	2176 nt	complete coding sequence	14.5767	**orf:** RdRp (pfam05919); translation table 4
seq170	PX098685	Teltow Canal mito-like virus 8 MR233-17E/30091	1124 nt	partial	10.9858	**orf:** RdRp (pfam05919); translation table 4
seq171	PX098686	Havel River narna-like virus 1 MR233-17E/712	2480 nt	partial	11.656	orf-partial: RdRp (cd23177)
seq172	PX098687	Havel River narna-like virus 2 MR233-17E/1038	2074 nt	partial	16.9147	**orf-par:** RdRp (cd23177)
seq173	PX098688	Havel River narna-like virus 3 MR233-17E/2282	1450 nt	partial	9.17862	**orf-par:** RdRp (cd23177); **rORF-par:** hypothetical protein
seq174	PX098689	Havel River narna-like virus 4 MR233-17E/520	2880 nt	complete coding sequence	85.9087	**orf:** RdRp (cd23177); **rORF-par:** hypothetical protein
seq175	PX098690	Teltow Canal narna-like virus 1 MR233-17E/5739	2605 nt	partial	271.948	**orf-par:** RdRp (cd23177); **rORF-par:** hypothetical protein
seq176	PX098691	Teltow Canal artivirus 1 MR233-17E/1135	5923 nt	partial	31.1241	**orf1-par:** structural protein; **orf2:** RdRp (pfam02123)
seq177	PX098692	Teltow Canal artivirus 2 MR233-17E/7091	4243 nt	partial	35.2168	**orf1-par:** structural protein; **orf2:** RdRp (pfam02123)
seq178	PX098693	Teltow Canal artivirus 3 MR233-17E/5591	2643 nt	partial	36.4487	**orf1-par:** structural protein
seq179	PX098694	Teltow Canal pseudototivirus 1 MR233-17E/1785	4752 nt	partial	32.9853	**orf1:** structural protein; **orf2-par:** RdRp (pfam02123)
seq180	PX098695	Teltow Canal pseudototivirus 2 MR233-17E/6580	2422 nt	partial	13.4277	**orf-par:** RdRp (pfam02123)
seq181	PX098696	Teltow Canal pseudototivirus 3 MR233-17E/7611	2245 nt	partial	24.7742	**orf-par:** RdRp (pfam02123)
seq182	PX098697	Teltow Canal pseudototivirus 4 MR233-17E/8790	2078 nt	partial	15.5115	**orf-par:** RdRp (pfam02123)
seq183	PX098698	Teltow Canal totivirus 1 MR233-17E/2398	4074 nt	partial	15.5746	**orf1-par:** hypothetical protein; **orf2-par:** RdRp (pfam02123)
seq184	PX098699	Teltow Canal totivirus 2 MR233-17E/4921	2822 nt	partial	30.545	**orf-par:** RdRp (pfam02123)
seq185	PX098700	Teltow Canal botybirna-like virus 1 MR233-17E/19434	1395 nt	partial	5.94265	**orf-par:** RdRp (pfam02123)
seq186	PX098701	Teltow Canal botybirna-like virus 2 MR233-17E/6210	2497 nt	partial	16.0364	**orf-par:** RdRp (pfam02123)
seq187	PX098702	Teltow Canal botybirna-like virus 3 MR233-17E/29853	1128 nt	partial	9.7961	**orf-par:** RdRp (pfam02123)
seq188	PX098703	Teltow Canal botybirna-like virus 4 MR233-17E/19538	1391 nt	partial	12.4637	**orf-par:** RdRp (pfam02123)
seq189	PX098704	Havel River toti-like virus 1 MR644-18E/87	7077 nt	partial	11.835	**orf1-par:** hypothetical protein; **orf2:** RdRp (pfam02123)
seq190	PX098705	Havel River toti-like virus 2 MR644-18E/89	6963 nt	(almost) complete genome	31.6345	**orf1:** hypothetical protein; **orf2:** RdRp (pfam02123)
seq191	PX098706	Havel River toti-like virus 3 MR644-18E/180	7060 nt	(almost) complete genome	19.9368	**orf1:** hypothetical protein; **orf2:** RdRp (pfam02123)
seq192	PX098707	Havel River toti-like virus 4 MR644-18E/700	2493 nt	partial	10.489	**orf1-par:** hypothetical protein; **orf2-par:** RdRp (pfam02123)
seq193	PX098708	Teltow Canal toti-like virus 1 MR233-17E/432	8814 nt	partial	59.8586	**orf1-par:** hypothetical protein; **orf2-par:** RdRp (pfam02123)
seq194	PX098709	Teltow Canal toti-like virus 2 MR233-17E/470	8631 nt	(almost) complete genome	168.413	**orf1:** hypothetical protein; **orf2:** RdRp (pfam02123)
seq195	PX098710	Teltow Canal toti-like virus 3 MR233-17E/533	8336 nt	(almost) complete genome	169.344	**orf1:** hypothetical protein; **orf2:** RdRp (pfam02123)
seq196	PX098711	Teltow Canal toti-like virus 4 MR233-17E/578	8072 nt	(almost) complete genome	65.0629	**orf1:** hypothetical protein; **orf2:** RdRp (pfam02123)
seq197	PX098712	Teltow Canal toti-like virus 5 MR233-17E/602	7975 nt	(almost) complete genome	114.369	**orf1:** hypothetical protein; **orf2:** RdRp (pfam02123)
seq198	PX098713	Teltow Canal toti-like virus 6 MR233-17E/603	7974 nt	(almost) complete genome	33.7126	**orf1:** hypothetical protein; **orf2:** RdRp (pfam02123)
seq199	PX098714	Teltow Canal toti-like virus 7 MR233-17E/670	7612 nt	partial	31.4276	**orf1-par:** hypothetical protein; **orf2:** RdRp (pfam02123)
seq200	PX098715	Teltow Canal toti-like virus 8 MR233-17E/683	7503 nt	partial	83.7934	**orf1-par:** hypothetical protein; **orf2:** RdRp (pfam02123)
seq201	PX098716	Teltow Canal toti-like virus 9 MR233-17E/684	7498 nt	(almost) complete genome	151.519	**orf1:** hypothetical protein; **orf2:** RdRp (pfam02123)
seq202	PX098717	Teltow Canal toti-like virus 10 MR233-17E/736	7207 nt	(almost) complete genome	93.8533	**orf1:** hypothetical protein; **orf2:** RdRp (pfam02123)
seq203	PX098718	Teltow Canal toti-like virus 11 MR233-17E/746	7161 nt	partial	122.419	**orf1-par:** hypothetical protein; **orf2:** RdRp (pfam02123)
seq204	PX098719	Teltow Canal toti-like virus 12 MR233-17E/762	7118 nt	partial	73.0524	**orf1-par:** hypothetical protein; **orf2:** RdRp (pfam02123)
seq205	PX098720	Teltow Canal toti-like virus 13 MR233-17E/815	6881 nt	(almost) complete genome	560.606	**orf1:** hypothetical protein; **orf2:** RdRp (pfam02123)
seq206	PX098721	Teltow Canal toti-like virus 14 MR233-17E/816	6877 nt	(almost) complete genome	90.1575	**orf1:** hypothetical protein; **orf2:** RdRp (pfam02123)
seq207	PX098722	Teltow Canal toti-like virus 15 MR233-17E/858	6723 nt	partial	22.7662	**orf1-par:** hypothetical protein; **orf2:** RdRp (pfam02123)

seq208	PX098723	Teltow Canal toti-like virus 16 MR233-17E/899	6584 nt	(almost) complete genome	167.386	**orf1:** hypothetical protein; **orf2:** RdRp (pfam02123)
seq209	PX098724	Teltow Canal toti-like virus 17 MR233-17E/929	6483 nt	(almost) complete genome	179.248	**orf1:** hypothetical protein; **orf2:** RdRp (pfam02123)
seq210	PX098725	Teltow Canal toti-like virus 18 MR233-17E/1193	6611 nt	(almost) complete genome	852.9	**orf1:** hypothetical protein; **orf2:** RdRp (pfam02123)
seq211	PX098726	Teltow Canal toti-like virus 19 MR233-17E/8568	2828 nt	partial	32.1853	**orf-par:** RdRp (pfam02123)
seq212	PX098727	Teltow Canal toti-like virus 20 MR233-17E/2149	4310 nt	partial	22.0912	**orf1-par:** hypothetical protein; **orf2:** RdRp (pfam02123)
seq213	PX098728	Teltow Canal toti-like virus 21 MR233-17E/2225	4243 nt	partial	35.8829	**orf1-par:** hypothetical protein; **orf2:** RdRp (pfam02123)
seq214	PX098729	Teltow Canal toti-like virus 22 MR233-17E/2420	4180 nt	partial	81.9404	**orf1-par:** hypothetical protein; **orf2-par:** RdRp (pfam02123)
seq215	PX098730	Teltow Canal toti-like virus 23 MR233-17E/2480	4108 nt	partial	101.825	**orf1-par:** hypothetical protein; **orf2:** RdRp (pfam02123)
seq216	PX098731	Teltow Canal toti-like virus 24 MR233-17E/2638	3888 nt	partial	123.95	**orf1-par:** hypothetical protein; **orf2:** RdRp (pfam02123)
seq217	PX098732	Teltow Canal toti-like virus 25 MR233-17E/2832	3761 nt	partial	220.638	**orf-par:** coat protein (pfam05518)
seq218	PX098733	Teltow Canal toti-like virus 26 MR233-17E/2981	6948 nt	(almost) complete genome	61.5379	**orf1:** hypothetical protein; **orf2:** RdRp (pfam02123)
seq219	PX098734	Teltow Canal toti-like virus 27 MR233-17E/3302	3494 nt	partial	52.8426	**orf-par:** RdRp (pfam02123)
seq220	PX098735	Teltow Canal toti-like virus 28 MR233-17D/198	7092 nt	partial	606.308	**orf1:** hypothetical protein; **orf2:** RdRp (pfam02123)
seq221	PX098736	Teltow Canal toti-like virus 29 MR233-17E/3703	6217 nt	partial	46.0655	**orf1-par:** hypothetical protein; **orf2-par:** RdRp (pfam02123)
seq222	PX098737	Teltow Canal toti-like virus 30 MR233-17E/4665	3625 nt	partial	29.0596	**orf-par:** RdRp (pfam02123)
seq223	PX098738	Teltow Canal toti-like virus 31 MR233-17E/4688	2892 nt	partial	22.8731	**orf:** RdRp (pfam02123)
seq224	PX098739	Teltow Canal toti-like virus 32 MR233-17E/4802	3079 nt	partial	21.3394	**orf-par:** RdRp (pfam02123)
seq225	PX098740	Teltow Canal toti-like virus 33 MR233-17E/4031	7493 nt	partial	32.777	**orf1-par:** hypothetical protein; **orf2:** RdRp (pfam02123)
seq226	PX098741	Teltow Canal toti-like virus 34 MR233-17E/5262	2729 nt	partial	30.8014	**orf:** RdRp (pfam02123)
seq227	PX098742	Teltow Canal toti-like virus 35 MR233-17E/5779	2595 nt	partial	15.7195	**orf-par:** RdRp (pfam02123)
seq228	PX098743	Teltow Canal toti-like virus 36 MR233-17E/9548	1995 nt	partial	5.20251	**orf-par:** RdRp (pfam02123)
seq229	PX098744	Teltow Canal toti-like virus 37 MR233-17D/9420	4424 nt	partial	41.0145	**orf1-par:** hypothetical protein; **orf2:** RdRp (pfam02123)
seq230	PX098745	Teltow Canal toti-like virus 38 MR233-17E/8336	2138 nt	partial	7.64359	**orf-par:** RdRp (pfam02123)
seq231	PX098746	Teltow Canal toti-like virus 39 MR233-17E/420	8874 nt		173.899	Numerous internal termination codons in any of the tested translation tables.
seq232	PX098747	Teltow Canal toti-like virus 40 MR233-17E/478	8579 nt		69.4081	Numerous internal termination codons in any of the tested translation tables.
seq233	PX098748	Teltow Canal toti-like virus 41 MR233-17E/506	8471 nt		133.412	Numerous internal termination codons in any of the tested translation tables.
seq234	PX098749	Teltow Canal toti-like virus 42 MR233-17E/536	8325 nt		143.959	Numerous internal termination codons in any of the tested translation tables.
seq235	PX098750	Teltow Canal carasco-like virus 1 MR233-17E/1029	6164 nt	partial	2866.05	**orf1-par:** Prot (pfam13365) - RdRp (cd23184); **orf2:** hypothetical protein; **orf3:** structural protein (pfam00729)
seq236	PX098751	Teltow Canal carasco-like virus 2 MR233-17E/1173	5830 nt	partial	54.6666	**orf1-par:** Prot (pfam13365) - RdRp (cd23184); **orf2:** hypothetical protein; **orf3:** structural protein (pfam00729)
seq237	PX098752	Teltow Canal carasco-like virus 3 MR233-17E/15109	1582 nt	partial	9.43932	**orf1-par:** Prot (pfam13365) - RdRp (cd23184); **orf2:** hypothetical protein
seq238	PX098753	Teltow Canal Sclerophthora macrospora B-like virus MR233-17E/1463	5226 nt	(almost) complete genome	438.597	**orf1:** Prot (COG3591) - RdRp (pfam00680); **orf2:** structural protein
seq239	PX098754	Teltow Canal noda-like virus 168 MR233-17E/12610	1736 nt	partial	16.4683	**orf-par:** MethylTr (pfam19222) - RdRp (cd23173)
seq240	PX098755	Teltow Canal noda-like virus 169 MR233-17E/13709	1662 nt	partial	11.1035	**orf1-par:** RdRp (cd23173); **orf2-par:** hypothetical protein
seq241	PX098756	Teltow Canal noda-like virus 170 MR233-17E/20677	1351 nt	partial	14.8571	**orf1-par:** RdRp (cd23173); **orf2-par:** hypothetical protein
seq242	PX098757	Teltow Canal noda-like virus 171 MR233-17E/21668	1322 nt	partial	13.3638	**orf1-par:** RdRp (cd23173)
seq243	PX098758	Teltow Canal noda-like virus 172 MR233-17E/27080	2789 nt	partial	7.30513	**orf1-par:** MethylTr (pfam19222) - RdRp (cd23173); **orf2-par:** hypothetical protein
seq244	PX098759	Teltow Canal noda-like virus 173 MR233-17E/28216	2895 nt	partial	121.214	**orf1-par:** MethylTr (pfam19222) - RdRp (cd23173); **orf2-par:** hypothetical protein
seq245	PX098760	Teltow Canal noda-like virus 174 MR233-17E/4755	2865 nt	partial	3513.53	**orf1-par:** MethylTr (pfam19222) - RdRp (cd23173); **orf2-par:** hypothetical protein
seq246	PX098761	Teltow Canal noda-like virus 175 MR233-17E/4771	2861 nt	partial	26.8937	**orf1-par:** MethylTr (pfam19222) - RdRp (cd23173); **orf2-par:** hypothetical protein
seq247	PX098762	Teltow Canal noda-like virus 176 MR233-17E/5098	2771 nt	partial	26.6716	**orf1-par:** MethylTr (pfam19222) - RdRp (cd23173); **orf2-par:** hypothetical protein
seq248	PX098763	Teltow Canal noda-like virus 177 MR233-17E/5111	2768 nt	partial	23.1423	**orf1-par:** MethylTr (pfam19222) - RdRp (cd23173); **orf2-par:** hypothetical protein
seq249	PX098764	Teltow Canal noda-like virus 178 MR233-17E/6711	2755 nt	partial	24.5016	**orf1-par:** MethylTr (pfam19222) - RdRp (cd23173); **orf2-par:** hypothetical protein
seq250	PX098765	Teltow Canal noda-like virus 179 MR233-17E/7722	2226 nt	partial	18.4699	**orf1-par:** MethylTr (pfam19222) - RdRp (cd23173); **orf2-par:** hypothetical protein
seq251	PX098766	Teltow Canal noda-like virus 180 MR233-17D/1963	2389 nt	partial	21.5839	**orf1-par:** MethylTr (pfam19222) - RdRp (cd23173); **orf2-par:** hypothetical protein
seq252	PX098767	Teltow Canal noda-like virus 181 MR233-17E/9259	2072 nt	partial	13.0164	**orf1-par:** RdRp (cd23173); **orf2-par:** hypothetical protein
seq253	PX098768	Teltow Canal noda-like virus 182 MR233-17E/10349	1916 nt	partial	7.60386	**orf-par:** MethylTr (pfam19222) - RdRp (cd23173)
seq254	PX098769	Teltow Canal noda-like virus 183 MR233-17D/126154	989 nt	partial	6.94944	**orf-par:** MethylTr (pfam19222)
seq255	PX098770	Teltow Canal noda-like virus 184 MR233-17E/5258	2730 nt	partial	346.656	**orf-par:** MethylTr (pfam19222) - RdRp (cd23173)
